# Computational analysis of speed-accuracy tradeoff

**DOI:** 10.1038/s41598-022-26120-2

**Published:** 2022-12-20

**Authors:** Marcin Penconek

**Affiliations:** Quantitative Psychology and Economics, University of Warsaw, Warsaw, Poland

**Keywords:** Computational biology and bioinformatics, Neuroscience, Psychology

## Abstract

Speed-accuracy tradeoff (SAT) in the decision making of humans and animals is a well-documented phenomenon, but its underlying neuronal mechanism remains unclear. Modeling approaches have conceptualized SAT through the threshold hypothesis as adjustments to the decision threshold. However, the leading neurophysiological view is the gain modulation hypothesis. This hypothesis postulates that the SAT mechanism is implemented through changes in the dynamics of the choice circuit, which increase the baseline firing rate and the speed of neuronal integration. In this paper, I investigated alternative computational mechanisms of SAT and showed that the threshold hypothesis was qualitatively consistent with the behavioral data, but the gain modulation hypothesis was not. In order to reconcile the threshold hypothesis with the neurophysiological evidence, I considered the interference of alpha oscillations with the decision process and showed that alpha oscillations could increase the discriminatory power of the decision system, although they slowed down the decision process. This suggests that the magnitude of alpha waves suppression during the event related desynchronization (ERD) of alpha oscillations depends on a SAT condition and the amplitude of alpha oscillations is lower in the speed condition. I also showed that the lower amplitude of alpha oscillations resulted in an increase in the baseline firing rate and the speed of neuronal intergration. Thus, the interference of the event related desynchronization of alpha oscillations with a SAT condition explains why an increase in the baseline firing rate and the speed of neuronal integration accompany the speed condition.

## Introduction

Despite decades of research on the speed-accuracy tradeoff (SAT), the underlying neuronal mechanism of controlling SAT in the brain remains unclear. SAT is the ability to adjust a response time to slow with high accuracy or fast with a high error rate^1^. This phenomenon was studied in humans^2–4^ and has been observed in other species, including primates, rodents, and insects^5^.

The last two decades of neurophysiological research have revealed the brain mechanism for decision making and shown that the formation of categorical decisions follows an integration-to-the-bound process^6–12^. The bounded integration framework was previously envisioned in system-level computational models such as the drift–diffusion model (DDM)^13,14^. In these models, SAT is conceptualized through the threshold hypothesis, which postulates that SAT is the result of adjustments to the decision threshold^15,16^: when speed is a priority, the decision threshold is decreased, and a decision is made faster based on integrating less information. When accuracy is a priority, the decision threshold is increased, leading to more information being accumulated at the expense of response time.

The stochastic variable describing the process of evidence accumulation in system-level models is interpreted as the firing rate of neurons performing neuronal integration process in vivo. Thus, it is expected that the firing rate at the moment of a decision varies between conditions (i.e., lower for speed priority and higher with an emphasis on accuracy). However, experimental evidence does not confirm this expectation. On the contrary, dot motion discrimination experiments show roughly the same firing rate at the threshold^8,9,17^. This evidence apparently challenges the threshold hypothesis as a possible implementation of SAT in the brain.

The causal mechanism of SAT and its implementation in the brain have been the subject of intensive scientific research over the last two decades^15–30^. As discussed by Standage and colleagues^27^, the alternative ways of implementing SAT can be linked to three main groups of neurophysiological mechanisms: modulation of evidence encoding, modulation of the integration of encoded evidence, and modulation of the amount of integrated evidence sufficient to make a decision.

Electrophysiological^17,24^ and neuroimaging experiments^18–20^ provide evidence that the baseline firing rates, the ramping rates of neuronal activity, and the hemodynamic response in regions of the brain implicated in decision making are higher in tasks that emphasize speed. These correlations suggest that SAT is controlled by mechanisms that affect the baseline firing rate and the speed of neuronal integration in the choice circuit. In the context of system-level decision-making models, these effects are linked to an increased baseline and drift rate in the speed condition, rather than to threshold adjustments. However, in many system-level models, changing the threshold is equivalent to changing the baseline^16^.

Computational mechanisms of SAT linked to modulation of the baseline firing rate and the speed of neuronal integration have been investigated^26,31–33^ using biophysically realistic decision-making model developed by Wang^34,35^. These investigations were instrumental in establishing the gain modulation hypothesis as the leading neurophysiological view of how SAT is controlled in the brain.

The gain modulation hypothesis^28^ assumes a fixed threshold and postulates that SAT is controlled by the excitability of neurons in the integrator circuit by non-selective excitatory and inhibitory inputs to the network performing evidence accumulation task. This increased excitability of neurons changes the dynamics of the decision-system and is manifested by an increased baseline firing rate and an increased speed of neuronal integration. Thus, the effects of modulation are consistent with the correlations uncovered in experimental research^17,24^. Because these effects are not restricted to the choice circuit only^24^, the hypothesis postulates a wider modulation involving sensory-encoding neurons with a top-down control signal.

However, the computational analysis supporting the gain modulation hypothesis is inconsistent with behavioral experiments. In experiments when accuracy is the priority, the mean reaction time (RT) on erroneous decisions is longer than that on correct decisions. While under the speed condition, the mean RT on erroneous decisions is shorter than that on correct decisions^36,37^. Wang’s model^34^ produces systematically longer mean decision times on erroneous than correct decisions^23,35^. Model predictions for the longer mean RTs on errors are inconsistent with the observed effects in the speed condition.

Furthermore, other neuronal mechanisms occurring before and during the decision process affect the decision-making speed and accuracy. Presentation of a stimulus or its anticipation leads to the desynchronization of alpha oscillations (i.e., event related desynchronization or ERD) followed by an increase in alpha power after task execution^38^. A larger value of alpha power during the anticipation of stimulus onset is positively correlated with performance^39–41^. Another experiment^42^ suggested that high alpha power in the anticipation period is related to longer decision time. All these results suggest that alpha oscillations might interfere with SAT effects.

A recent behavioral experiment conducted by Rafiei and Rahnev^43^ uncovered non-linear relationships in behavioral response to varying SAT conditions, which can shed light on how SAT is implemented in the brain. In the current paper, I re-examined alternative computational mechanisms of SAT and showed that the threshold hypothesis was qualitatively consistent with their experimental data, whereas the gain modulation hypothesis^28^ was not. I also showed that the interference of alpha oscillations with the decision process could explain the apparent inconsistency of the threshold hypothesis with neurophysiological evidence.

**Figure 1:**
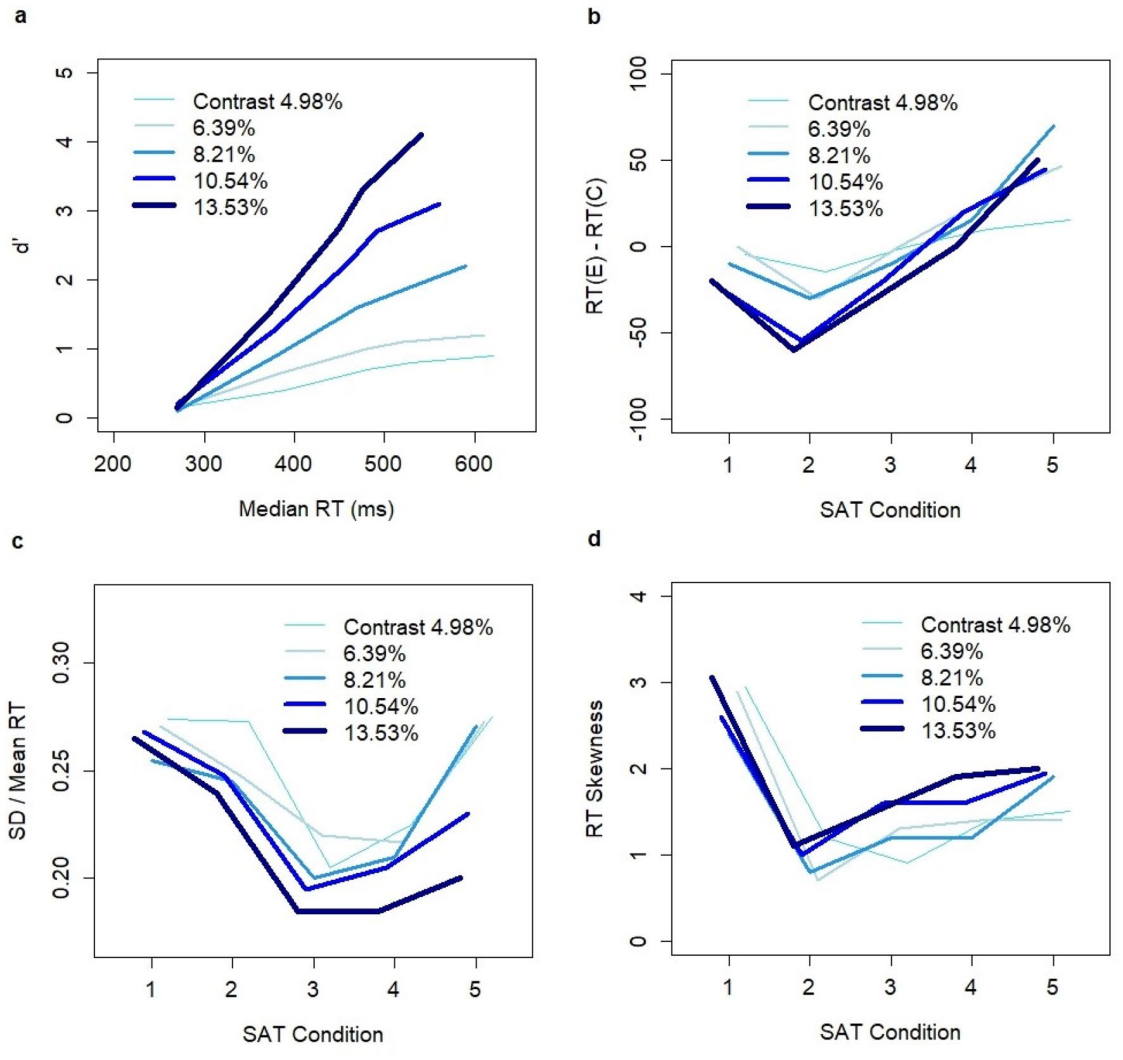
Experimental data of Rafiei and Rahnev ^43^. (**a**) Relationship between median RT and d’ for five SAT conditions and five contrast levels where d’ = Φ^−1^(correct response rate)–Φ^−1^(erroneous response rate) and Φ^−1^ is the inverse of the cumulative normal distribution N(0,1). The effects of five SAT conditions and five contrast levels on (**b**) the difference in mean RT between erroneous and correct decisions, (**c**) SD/mean RT ratio, and (**d**) RT skewness. This figure was adapted from Fig. 3 in ^43^ with authors’ permission. In the adaptation, I show the average estimates only (i.e., without reporting error terms) and provide a new figure description. The original figure was featured in the paper ^43^ published in Scientific Reports under Creative Commons Attribution 4.0 International License; https://creativecommons.org/licenses/by/4.0/legalcode.

The alternative computational mechanisms of the SAT are investigated based on the experimental data of Rafiei and Rahnev^43^. The experiment was conducted on human subjects and involved the manipulation of time-pressure through a reward scheme by linking the levels of incentives to response latency. The task was to identify the direction of a Gabor patch presented to subjects at an angle of + 45 or − 45°. Task difficulty was controlled by the contrast of the patch. Five SAT levels and five contrast levels were considered. The authors analyzed the impact of SAT condition on the difference in mean RTs between erroneous and correct decisions, the ratio of the standard deviation and mean RT, and the skewness of RT distributions. The experiment discovered a robust U-shape relationship between SAT levels and the difference in RT between erroneous and correct trials (Fig. 1b). When accuracy was a priority, mean RTs on erroneous decisions were longer than those on correct decisions (except for the lowest contrast level, Fig. 1b). I will refer to this pattern of RTs as the default RT pattern. When speed was a priority, mean RTs on erroneous decisions were shorter than those on correct decisions (except for the lowest contrast level). I will refer to this pattern of RTs as the reverse RT pattern.

These results were consistent with previous observations in behavioral studies on human subjects. As speed became strongly emphasized, the difference between RTs approached zero (i.e., the mean RT for erroneous decisions and that for correct decisions became roughly equal). These results made intuitive sense because the decisions were made almost at random when speed was strongly emphasized. Thus, no difference in mean RTs was observed between the erroneous and correct trials. The described effects produced a U-shape relationship between the difference in RTs for erroneous and correct trials and the SAT condition. Similar U-shape relationships were observed between SAT condition and SD/mean RT ratio (Fig. 1c) and the skewness of RT distributions (Fig. 1d).

Experimental data were fitted with the drift diffusion model (DDM). The authors showed that the model could not replicate the observed patterns under the selective influence assumption that SAT condition was adjusted by changes in the threshold value. The uncovered non-linear relationships could pose a challenging task for decision-making models, and the authors suggested that the results be used as a validation test for decision-making models. In response to the Rafiei and Rahnev experiment, Ratcliff and Kang developed a mixture model incorporating the DDM with random fast guesses represented by a normal distribution of fixed mean and standard deviation^44^. The model was shown to fit the data from the experiment.

The current paper continues the discussion of SAT initiated by Rafiei and Rahnev^43^ and is a response to their experimental findings. The aim of this paper was to shed light on how the SAT mechanism is implemented in the brain by investigating alternative computational mechanisms of SAT. These alternative mechanisms were studied with the use of mechanistic decision-making model introduced by the author^45^. This model implements the non-linear neuronal integration process as an emergent property of the attractor network with binary neurons. However, the impact of the current paper does not depend on the results of Ratcliff and Kang^44^, which used a system-level decision-making model to fit the experimental data. A system-level approach has been successful in modeling many behavioral experiments, but it is insufficient to distinguish between alternative computational mechanisms of SAT, as argued by Bogacz and colleagues^16^. In this paper, we used a mechanistic approach that relied on simulating populations of neurons.

## Results

**Figure 2:**
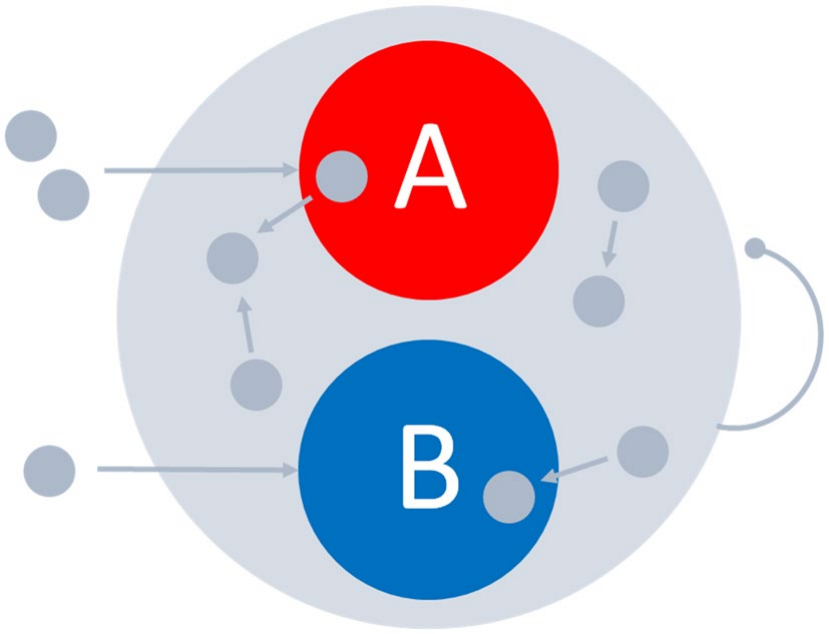
Network architecture of the decision system: the recurrent network of the excitatory neurons has a higher density of connections in neuronal pools A and B. Neuronal pools A and B form the decision sets and are selective to inputs. Inputs are provided by separate pools of neurons (outside of the decision system) projecting to A and B, respectively. The system is equipped with a global inhibition mechanism that facilitates winner-take-all competition.

The decision system consists of three populations of excitatory neurons with random recurrent connections: decision pools A, B, and the rest of the network (Fig. 2).

Decision pools A and B are selective to inputs. The strength of inputs is controlled by the time-variable activity of additional pools of neurons (outside of the choice circuit) that encode evidence in favor of each decision alternative and deliver the input to respective subsets of neurons in A and B. The activity follows a Poisson distribution with parameters denoted by *λ*_*A*_ and *λ*_*B*_. These parameters are responsible for the drift rate of integration by increasing the excitatory postsynaptic input of neurons in parallel to the input provided through the recurrent connections. The circuit is equipped with a global inhibition mechanism ensuring the stability of the network and facilitating winner-take-all-competition between the decision pools A and B. The global inhibition mechanism is implemented through a common activation threshold. The activation threshold reflects the inhibition level and is a function of currently active neurons. It is parameterized with the inhibition constant *Θ*. The inhibition constant controls the excitability of neurons in the decision circuit (i.e., higher values of *Θ* increase the likelihood of passing the activation threshold). As we shall see, higher values of *Θ* increase the baseline firing rate of neurons in the circuit and decrease the decision time. The decision variable is defined in terms of the population-average firing rate of neurons in either pool A or pool B that is winning the competition and compared to the predefined decision threshold value. The above parameters (i.e., *λ*_*A*_, *λ*_*B*_, *Θ*, and the decision threshold) are manipulated when considering alternative mechanisms of SAT. Other model parameters control the global behavior of the system and are kept constant at their default values. Please refer to Methods for more details.

The system has three semi-stable states: the spontaneous state in which neurons fire with a low firing rate (4–20 Hz) and two decision states in which neurons in either A or B (but not both) fire with a high firing rate (51–66 Hz). When inputs are provided, the system facilitates the neuronal integration process from the spontaneous state (starting point of all simulations) to one of the decision states.

The model dynamics of the neuronal integration process resemble the dynamics of the leaky competing accumulator model^46^. The leaky competing accumulator model by Usher and McClelland is a system-level model and its construction was inspired by neurophysiological considerations. This model can be a useful analogy for our mechanistic model. However, it is unclear whether our model can be described by the system of stochastic equations postulated by the leaky competing accumulator model.

Standage and colleagues^27^ discussed three groups of possibilities for the neurophysiological mechanism of SAT: modulation of the amount of integrated evidence sufficient to make a choice, modulation of evidence encoding, and modulation of the integration of encoded evidence.

In this paper, I was interested in considering alternative ways of implementing the SAT mechanism from a computational perspective. Within this context, I considered three possibilities: (1) modulation of the decision threshold, which I refer to as the threshold hypothesis, (2) modulation of inputs, which I refer to as the evidence encoding hypothesis, and (3) modulation of the control parameters responsible for the excitability of neurons in the choice circuit, which I refer to as the gain modulation hypothesis^28^.

The evidence encoding hypothesis refers to any mechanism involving changes to the decision circuit inputs including the possibility of additional sources of inputs not related to evidence encoding. The convention used in this paper borrowed from the categorization suggested by Standage and colleagues^27^ and operationalizes it from the computational perspective of the current model.

**Figure 3:**
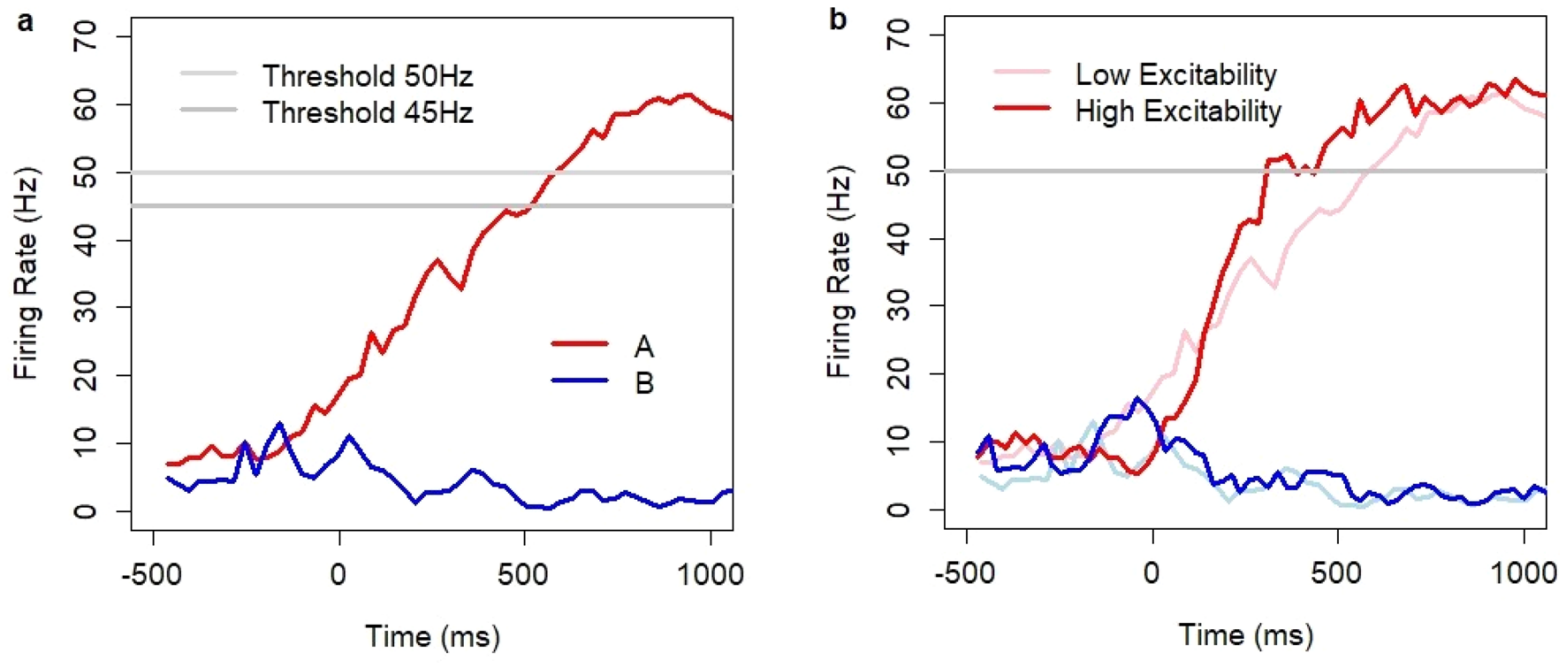
Modulation of the decision threshold (**a**) and the excitability level (**b**) and the effect on the neuronal integration process performed by the attractor network with decision pools A and B, which encode the categorical decision.

The effects of the two key alternative hypotheses (i.e., the threshold hypothesis and the gain modulation hypothesis) are illustrated in Fig. 3.

The well-documented non-linearity of the neuronal integration process^47,48^ suggests that the effects of each type of modulation can be different. Intuitively, although changes to inputs and the parameters controlling the excitability of neurons are likely to speed up and rescale the neuronal integration process without affecting its intrinsic properties, changes to the decision threshold would lead to termination of the process before it reaches the plateau of the decision state. Because the integration process is non-linear, termination of the process before it reaches the decision state can affect its intrinsic properties. As we shall see, one such property is the ability of the decision system to produce a reverse RT pattern (i.e., shorter RTs on erroneous decisions than on correct ones).

Let us start with the threshold hypothesis and show that the results of model simulations based on this hypothesis were qualitatively consistent with the experimental data.

### Threshold hypothesis

The hypothesis was modeled by assuming a linear relationship between stimulus and inputs to the decision system. The model was simulated with the Poisson inputs *λ*_*A*_ and *λ*_*B*_ (sampled every 30 ms) depending on the contrast level c = 4.98, 6.39, 8.21, 10.54, or 13.53%:

$${\lambda }_{A}= {\lambda }_{0}(\frac{1}{2}+ c)$$

$${\lambda }_{B}= {\lambda }_{0}(\frac{1}{2}- c)$$

where *λ*_*0*_ = 15. Inputs reflected both the evidence in favor of each direction (i.e., + 45 or − 45°) and the evidence against, which were assumed to be provided by sensory neurons specific to each of these directions. To simplify the simulations stronger evidence was always provided to decision set A. The results were based on N = 15,000 simulations (3000 per contrast level) using standard values of the control parameters. I considered threshold parameters in the range of 20–50 Hz. The lowest value (20 Hz) was the upper fuzzy border of the spontaneous state, whereas the highest value (50 Hz) was the bottom fuzzy border of the decision state. Thus, the threshold parameters reflected the intermediate values of the neuronal integration process along the trajectory from the spontaneous state to the decision state. The decision variable was defined in terms of the population-average firing rate in the winning decision pool of neurons (i.e., regardless of the firing rate in the losing pool) and was compared to the threshold parameter to determine the RT. The firing rate was calculated based on 30-ms lags sampled every 10 ms. Each model simulation started with 1000 ms of spontaneous model run before stimulus onset. No-decisions or decisions that took longer than 2000 ms were removed before calculating the relationship between median RT and d’ and the analysis of RT difference (Rafiei and Rahnev excluded the decisions which took shorter than 150 ms or took longer than 1500 ms). No restriction to 2000 ms was used for calculating the SD/mean RT ratio or the skewness of RT distributions. No non-decision time was assumed.

**Figure 4:**
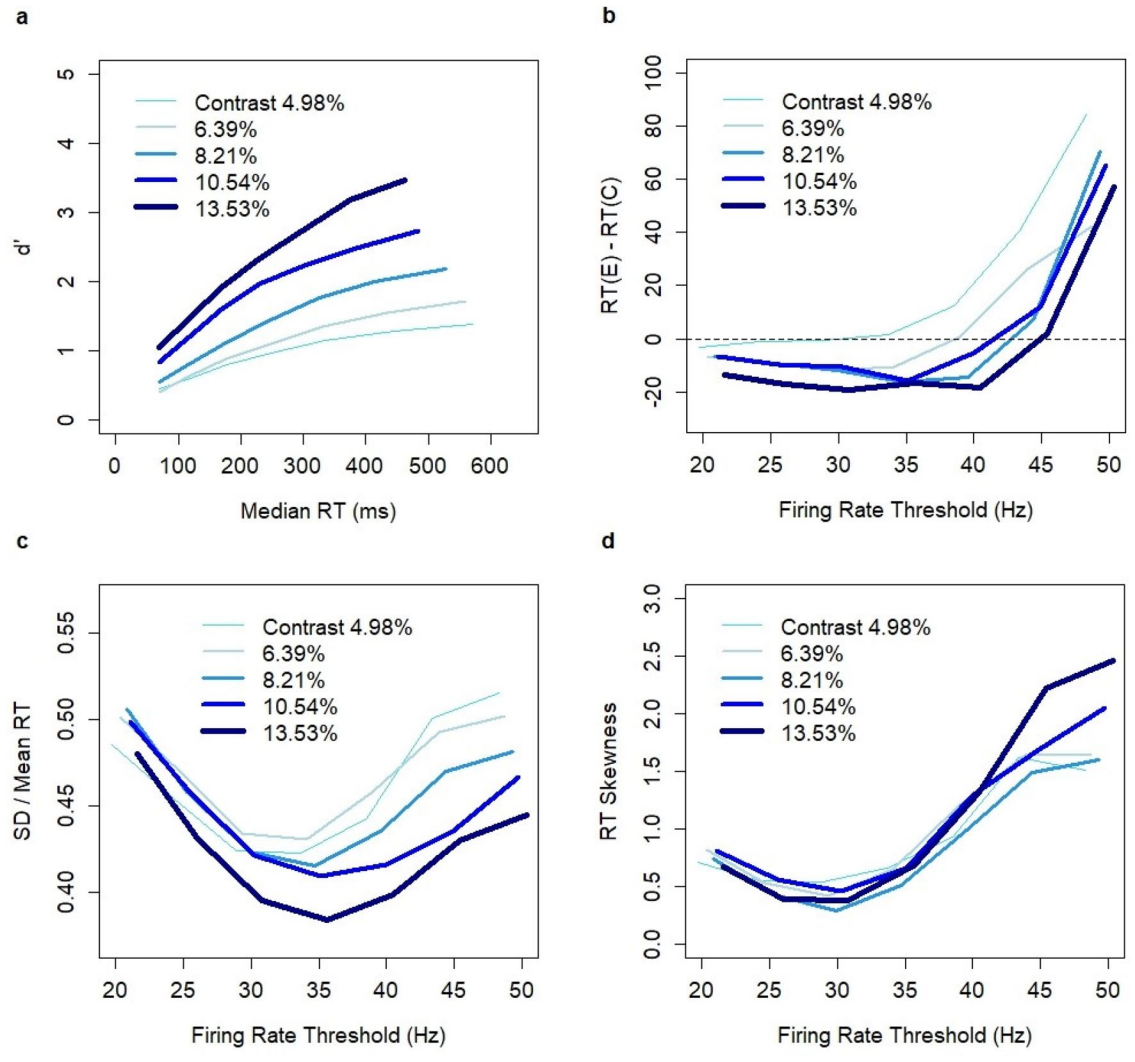
Model predictions based on N = 15,000 simulations (3000 per contrast level) with SAT effects implemented through a population-average firing rate threshold. Each data point reflects a different threshold level: 20, 25, 30, 35, 40, 45, or 50 Hz. (**a**) Speed-accuracy tradeoff for five contrast levels (4.98–13.53%). (**b**) Difference between RT (ms) on erroneous and correct decisions as a function of firing rate threshold (range: 20–50 Hz) in the winning pool. (**c**) Ratio between standard deviation and mean RT. (**d**) Skewness of the RT distribution. No non-decision constant was assumed.

The results of model simulations (Fig. 4) were qualitatively consistent with the experimental data. The relationships between RT and accuracy (d’) reflected manipulations in the SAT conditions that were achieved in the experiment (Fig. 1a). Median RTs in model simulations were systematically shorter than those in the experiment (Fig. 1a) suggesting the non-decision time in the range of 100–150 ms. Non-decision time is typically used in modeling behavioral experiments, and its justification assumes the existence of cognitive processes that have no effect on the decision process but that do affect the RT. On the neurophysiological level, it is assumed that additional time is needed to encode sensory evidence and execute an action. Although a constant non-decision time affects the median RT, it has no impact on the RT difference between correct and erroneous decisions, the standard deviation, or the skewness of RT distributions.

The model produced a reverse RT pattern with low-frequency thresholds (20–35 Hz) across all contrast levels (except for the lowest contrast level). This conclusion was confirmed by bootstrap estimates of the confidence intervals for the RT difference between erroneous and correct decisions (Appendix, Table 1). The U-shape relationship was replicated as the difference approached zero at the lowest SAT levels (Fig. 4b). Both the relationship between the frequency thresholds and SD/mean RT ratio and the skewness of RT distributions formed U-shape patterns (Fig. 4c and 4d) consistent with the experimental results. Note that the skewness of RT was the highest for the “high speed” condition in the experiment of Rafiei and Rahnev (Fig. 1d), whereas our computational analysis indicated only a slight uplift of skewness for the low threshold levels (20–25 Hz).

Other hypotheses assume that the threshold value is hard-wired and fixed. The fitness maximization principle suggests that the threshold value should allow the decision system to achieve its maximum accuracy. Any value below the threshold associated with maximal accuracy would permanently impair an animal’s ability to make correct decisions. This suggests that the threshold should fall within the basin of attraction of the decision state. However, the borders of the basin of attraction sets were fuzzy (Fig. 9b in Methods). Initial conditions with firing rates below 45–50 Hz could still lead to convergence to the spontaneous state, and the system kept improving the accuracy along with higher threshold levels. This excluded threshold values below 50 Hz and suggested the threshold value within the interval defined by the boarders of the decision state. A reasonable choice of the threshold aligns it with the effective lower border of the decision state (i.e., 50 Hz) because any higher threshold would lead to longer RTs with diminishing further improvement of accuracy.

### Evidence encoding hypothesis

Let us now consider the hypothesis that SAT is controlled by changes in the inputs. If this hypothesis is true, the system should be able to produce the reverse RT pattern for some range of input values. This was not the case. RTs on errors were longer than those on correct decisions for the full range of inputs to the decision system.

This conclusion was based on a Monte Carlo analysis of N = 20,000 model simulations with random uniformly distributed values of the Poisson parameters of *λ*_*A*_ and *λ*_*B*_ in the range from 0 to 20. The model was simulated with standard values of the control parameters and a threshold value of 50 Hz.

**Figure 5:**
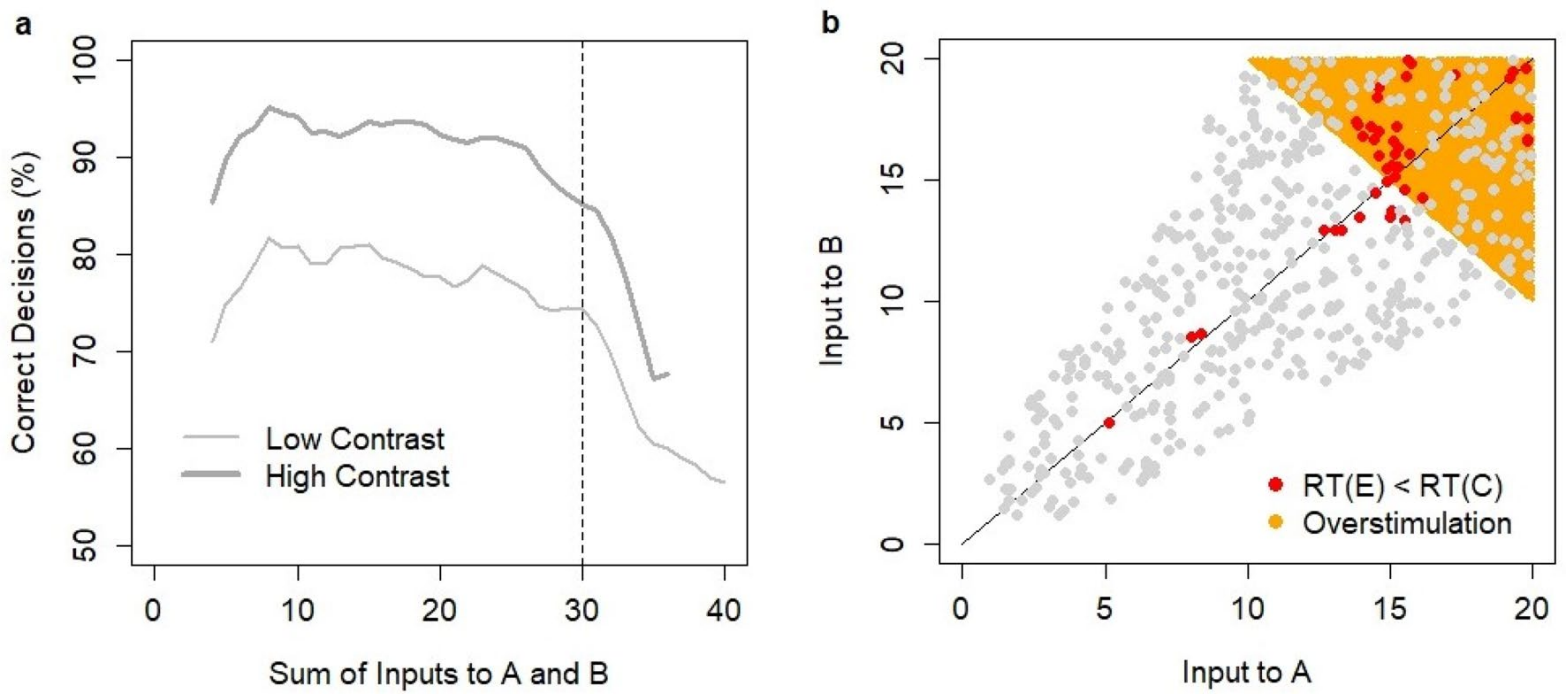
Model predictions based on N = 20,000 simulations with random values of Poisson inputs with parameter values *λ*_*A*_ and *λ*_*B*_ in the range 0–20. (**a**) Correct decisions as a function of the sum of inputs. (**b**) RT difference between erroneous and correct decisions based on a Monte Carlo analysis of the input space. Grey dots indicate the neighborhood when RTs on errors were longer than those on correct decisions. Red dots indicate the neighborhood when the opposite relationship held. The neighborhood (of maximum size ± 2.5) was set to contain at least 50 instances of erroneous decisions (a lower number of errors did not ensure enough stability). Otherwise, the point was not reported. The mean size of the neighborhood containing 50 errors was ± 1.43.

Notice that the model saturated at a high sum of input values. The percentage of correct decisions dropped when the sum of inputs exceeded 30 (Fig. 5a). Hence, a high sum of inputs could lead to overstimulation of the model. RT difference was tested by choosing a random point on the input space (two-dimensional uniform distribution) and comparing mean RTs on erroneous and correct decisions for simulations with input parameters in the neighborhood of this point. The reverse RT pattern (i.e., shorter RTs on erroneous decisions than those on correct ones; RT(E) < RT(C) in Fig. 5b) were located near the diagonal or in the overstimulation zone. Otherwise, the model produced the default RT pattern. This excluded the possibility of replicating the experimental results with any SAT mechanism based on a modulation of inputs (including an additional non-evidence related input).

### Gain modulation hypothesis

This hypothesis assumes that the threshold firing rate is hard-wired and fixed and SAT is controlled by a top-down control signal that projects non-selectively to sensory-encoding and integrator populations. This signal increases the excitability of neurons in the choice circuit, which increases the baseline firing rate and the speed of neuronal integration^28^. Because changes in sensory encoding cannot produce a reverse RT pattern, I focused on considering changes that affected the excitability of neurons in the choice circuit.

The excitability of the network in the model is controlled by the inhibition constant *Θ*, which sets the normative level of neuronal excitation (default value *Θ* = 0.13). An increase in the inhibition constant (*Θ* > 0.13) increases the level of neuronal excitation and is associated with the “speed” condition, whereas a decrease is associated with the “accuracy” condition. The domain of the parameter is the interval (0.1–0.2), which is related to the chosen proportion of neurons in the decision sets in the network (i.e., 10%). Inhibition levels below 0.1 would restrict the possibility of all neurons in the decision set from being active at the same time. Inhibition levels above 0.2 could lead to violating the winner-take-all property because it would allow neurons in both decision sets to be active at the same time.

**Figure 6:**
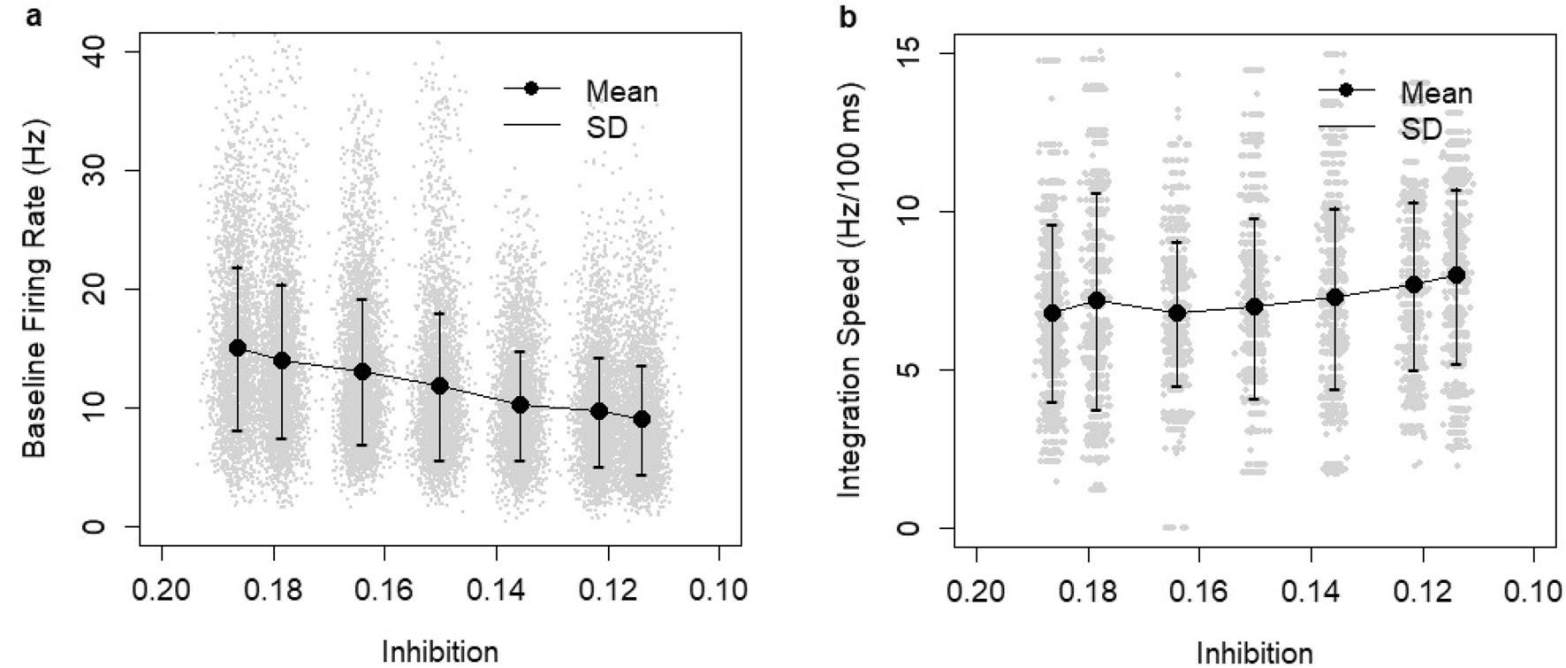
Effects of the manipulation of the inhibition constant. (**a**) Baseline firing rate (Hz): average in the decision set during the spontaneous run. (**b**) Integration speed (Hz/100 ms): average based on N = 7000 model runs with random contrast levels: 4.98–13.53% (i.e., N = 1000 per inhibition level).

Manipulations of the inhibition constant (0.1 < *Θ* < 0.2) led to changes in the baseline firing rate in the decision set in the spontaneous state that were consistent with the mechanism postulated by the gain modulation hypothesis (Fig. 6a). An increase in the inhibition constant caused an increase in the baseline firing rate (linear regression, slope = 81.4, t = 55.009, *p *< 2e^−16^; bootstrap estimate of slope: 81.42 ± 1.53, 95% confidence interval: (78.46, 84.42), N = 10,000 draws with replacement). In addition, an increase in inhibition constant decreased the RT but did not increase the integration speed (Fig. 6b). The reverse relationship held (linear regression, slope = − 13.5, t = − 9.61, *p *< 2e^−16^; bootstrap estimate of slope: − 13.47 ± 1.42, 95% confidence interval: (− 16.31, − 10.73), N = 10,000 draws with replacement). The above results were based on 7000 simulations with random values of contrast in the range from 4.98 to 13.53% (uniform distribution).

In order to understand the impact of manipulating the inhibition constant on SAT effects, I conducted N = 40,000 simulations (8000 per contrast level) with random values of the inhibition constant *Θ* in the range 0.1 to 0.2 (uniform distribution) and standard values of the other control parameters. A larger sample size of simulations and a randomized approach were used to study whether the experimental relationships could potentially hold on any interval within the domain of the parameter *Θ*. In line with the gain modulation hypothesis, which postulates a hard-wired threshold, the threshold was fixed and set to the value of 50 Hz (border of the decision set). I assumed the same linear relationship between stimulus and the Poisson inputs *λ*_*A*_ and *λ*_*B*_ as in the threshold hypothesis.

**Figure 7:**
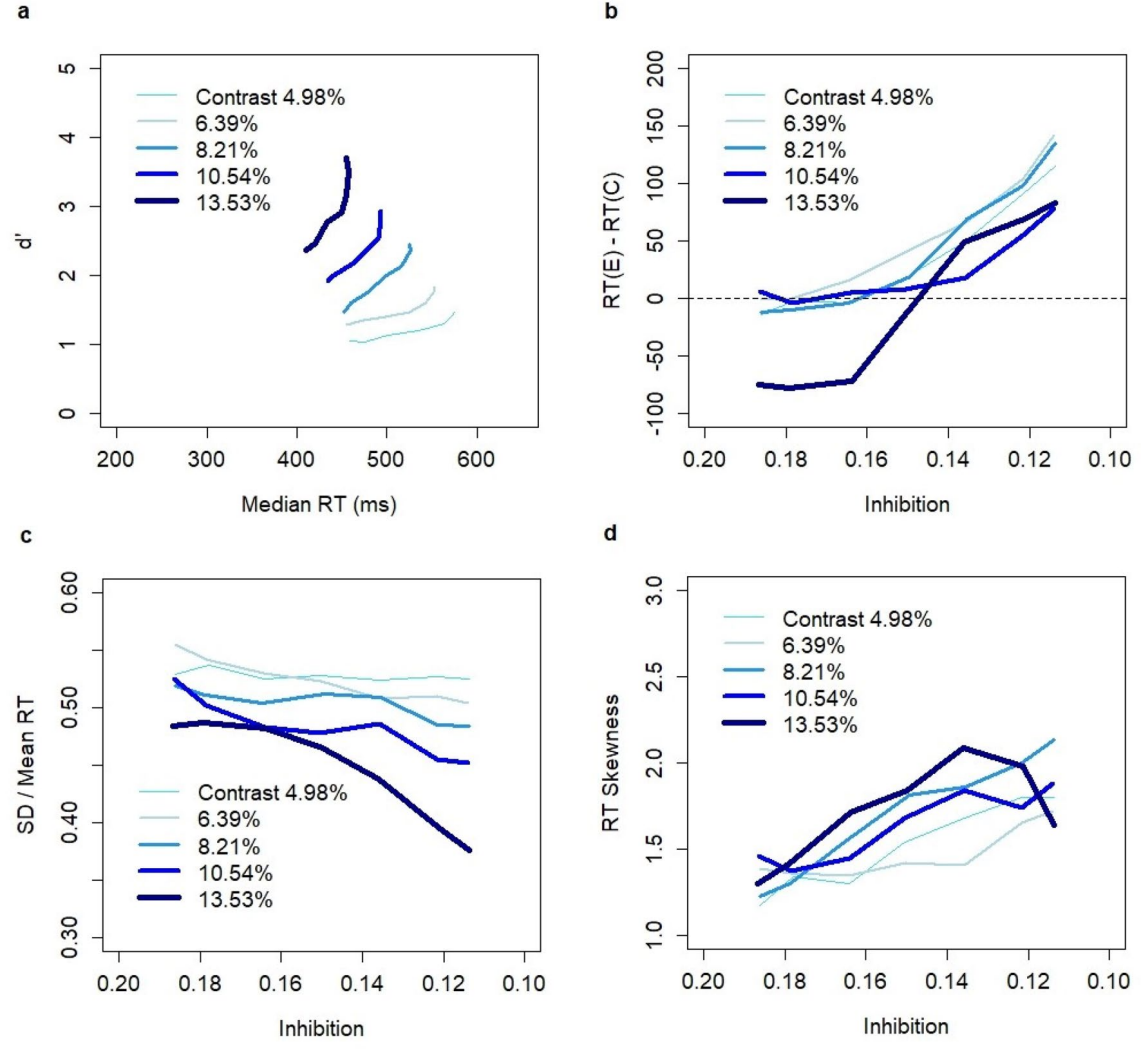
Model predictions based on N = 40,000 simulations (8000 per contrast level) with SAT effects implemented by manipulation of the inhibition constant (0.1–0.2). A higher inhibition constant indicates higher excitability of neurons in the system and, thus, is associated with the “speed” condition. (**a**) Speed-accuracy tradeoff for five contrast levels (4.98–13.53%). (**b**) Difference between RTs (ms) of erroneous and correct decisions as a function of inhibition constant (0.1–0.2). (**c**) Ratio between the standard deviation and mean RT. (**d**) Skewness of the RT distribution. No non-decision constant was assumed.

Changes in the inhibition constant created SAT effects (Fig. 7a) across all contrast levels. However, the patterns of SAT effects were not consistent with the data from the Rafiei and Rahnev experiment^43^. First, the magnitude of the effect on median RT (Fig. 7a) was much smaller than observed experimentally (Fig. 1a). It was clear that manipulations of the inhibition constant could not lead to larger effects, since the simulations covered the entire domain of reasonable parameter values and no non-decision time was assumed. Second, there were no reverse RT patterns in model simulations except for the highest contrast level. This result was confirmed by bootstrap estimates of the confidence intervals, which showed no evidence for shorter RTs on erroneous than correct decisions (Appendix, Table 2). It was confirmed that the shorter RTs on erroneous decisions than correct ones for the highest contrast level were related to a displacement of the decision state. Apparently, the borders of decision states were not constant. They became less coherent due to changes in the inhibition constant and moved with the strength of inputs. Third, there was no evidence of a U-shape relationship either for the RT difference (Fig. 7b), the SD/mean RT ratio (Fig. 7c), or the skewness of RT distributions (Fig. 7d). Hence, the model simulations show that the hypothesis that the computational mechanism behind SAT relies on the modulation of neuronal excitability in the choice circuit, which affects the baseline firing rate and the speed of the integration process, is inconsistent with the results of the experiment of Rafiei and Rahnev.

The above analysis provided support for the threshold hypothesis. However, it did not resolve the controversy with neurophysiologic evidence, which shows a higher level of baseline firing rates and a higher speed of neuronal integration in the speed condition^17,24^. If these effects are not responsible for SAT, they could be related to other neuronal mechanisms interfering with SAT. In the last part of the paper, I investigate the effects of desynchronization of alpha oscillations on the decision process. The purpose of this section is not to provide an explanation of the desynchronization mechanism itself but to show that alpha oscillations interact with the SAT mechanism. This interaction provides an alternative explanation of why the speed condition is accompanied by an increase in the baseline firing rate and the speed of neuronal integration. These investigations allow us to reconcile the threshold hypothesis with the neurophysiological evidence.

### Interference with alpha waves

A growing body of research shows that alpha oscillations interfere with the decision process and behavioral performance. Presentation of a stimulus or its anticipation leads to desynchronization of alpha oscillations (i.e., ERD) followed by alpha power increase after task execution^38^. The magnitude and timing of alpha waves suppression and resynchronization depend on the task type, duration, and difficulty. Large alpha power during the anticipation of stimulus onset is positively correlated with performance^39–41^. Evidence for the relationship between alpha power and decision time comes from the experiment of Paluch, Jurewicz, and Wróbel^42^, who analyzed responses among groups of participants responding fast or slowly to a decision task and showed that fast responders had a lower alpha power during the anticipation period. Another experiment^49^ showed that the effect of a large alpha power before stimulus onset might depend on task difficulty: longer decision times for an easy task and lower rates of erroneous responses for a difficult task. Experiments on monkeys performing somatosensory discrimination tasks with a sequential presentation of stimuli indicated that a decrease in alpha power (in premotor and motor regions) correlated with better discrimination performance^50^. The same study showed that spike activity was negatively correlated with alpha power and was phase dependent. The link between metabolic activity and alpha power has been reported in several studies^51–54^: an increase in alpha power is related to a decrease in metabolic rate. However, in some brain areas, the relation was reversed^51–55^.

### Model predictions

**Figure 8:**
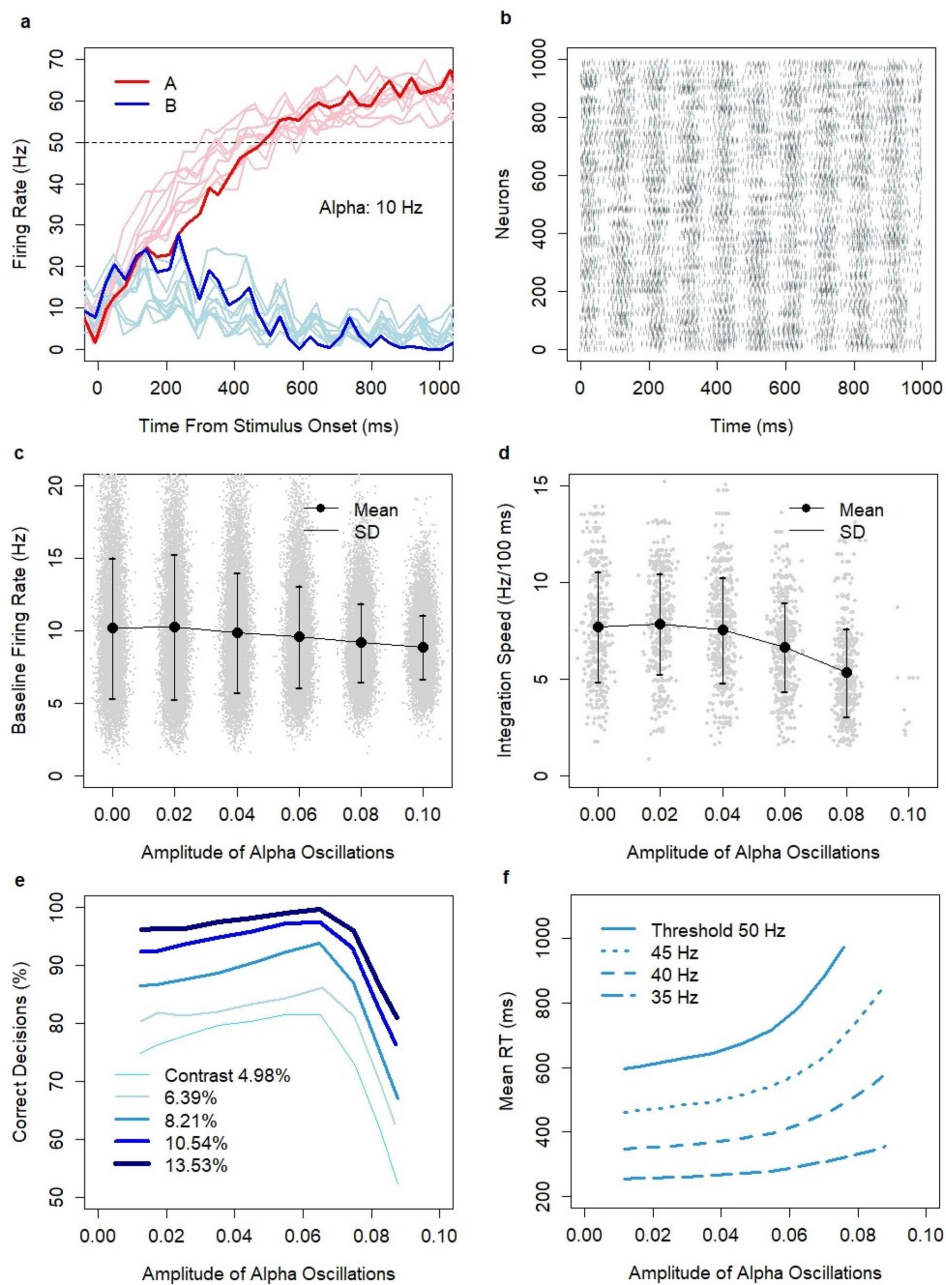
Interference between alpha oscillations (8–12 Hz) and the decision process. (**a**) Firing rate after stimulus onset in pools A and B for model runs with alpha oscillations (10 Hz) and coherence level c = 51.2% (where coherence describes task difficulty in dot motion discrimination experiments, see Methods). (**b**) Spiking activity of neurons in the decision system during a spontaneous model run with alpha oscillations (10 Hz). (**c**) Baseline firing rate (Hz): average in the decision set during a spontaneous run with alpha oscillations of random frequency (8–12 Hz); time bins 100 ms. (**d**) Integration speed (Hz/100 ms): average based on N = 2351 model runs with alpha oscillations of random frequency (8–12 Hz) and random contrast levels: 4.98–13.53%; time bins 30 ms. (**e**) Discriminatory power as a function of the amplitude of alpha oscillations (8–12 Hz) for different contrast levels and threshold 50 Hz. (**f**) Reaction time (RT) as a function of the amplitude of alpha oscillations (8–12 Hz) for different thresholds (35–50 Hz) and medium contrast level 8.21%.

By following the suggestions from the study of Haegens and colleagues^50^, I designed a computational experiment in which alpha oscillations were implemented through an inhibitory mechanism. The mechanism was exogenous to the decision system and was introduced through time-dependent oscillatory modulation of the inhibition constant:

$$\it \Theta ={ \Theta }_{0}+\alpha* \mathrm{sin}(\omega t+ \xi )$$

where *Θ*_*0*_ = 0.13 is the baseline inhibition constant, and *α*, *ω*, and *ξ* are the amplitude, frequency, and phase of alpha waves, respectively. Thus, neuronal excitability followed rhythmic changes. Whether a particular neuron was activated or not depended on the same update rule as before, except that the global threshold level was time-dependent. Changes with the same constant amplitude were applied throughout the entire model run including the pre-decision time and neuronal integration time. I considered alpha oscillations in the frequency range of 8–12 Hz and amplitudes in the range 0–0.1. In particular, for *α* = 0.1, the inhibition constant varied over time between 0.03 and 0.23. The initial phase *ξ* was a random parameter (uniform distribution in the range 0–2π). The other control parameters were set at their default values. The introduction of alpha oscillations did not compromise the fundamental properties of the model. The model kept the persistent activity of the network, exhibited the emergent neuronal integration process (Fig. 8a), and facilitated winner-take-all competition between the decision states. The activity of the network showed characteristic phase dependencies with a rhythmic increase and decrease in neuronal activity (Fig. 8b).

Let us now discuss how alpha oscillations impacted the baseline firing rate and the speed of neuronal integration. Despite symmetric changes in relation to the baseline inhibition constant, the baseline firing rate in the spontaneous state decreased as a function of amplitude (Fig. 8c; linear regression, slope = − 14.6, t = − 30.52, *p *< 2e^−16^; bootstrap estimate of slope: − 14.59 ± 0.48; 95% confidence interval: (− 15.55, − 13.65); N = 10,000 draws with replacement). Higher amplitudes also decreased the range of fluctuations of the network in the spontaneous state. The standard deviation was in the range of 4.8 and 5.0 for amplitude levels 0 or 0.02 and dropped to 2.7 and 2.2 for amplitude levels 0.08 or 0.1, respectively. These results were consistent with the experiments, which show a decrease in spike activity^50^ and metabolic rate^51–54^ with increased alpha power. Model predictions showed a negative relationship between alpha oscillations and the speed of the integration process (Fig. 8d) in the amplitude range of 0.02–0.08 (linear regression, slope = − 30.2, t = − 15.93, *p *< 2e^−16^; bootstrap estimate of slope: − 30.23 ± 1.90; 95% confidence interval: (− 33.94, − 26.54); N = 10,000 draws with replacement). The analysis was conducted with random contrasts (uniform distribution) in the range of 4.98–13.53%. Simulations with correct decisions were selected from the set of N = 3000 (500 per amplitude level) model simulations. The rate of no-decisions was high for the amplitude level of 0.1, resulting in only 11 correct decisions out of 500 for the highest amplitude value (*α* = 0.1).

The model also predicted the interference effects between alpha oscillations and the discriminatory power and the mean RT of the decision system. The analysis was conducted based on N = 15,000 model simulations for five contrast levels (3000 simulations per contrast level). Alpha oscillations of random frequency in the range of 8–12 Hz (uniform distribution), a random amplitude in the range of 0–0.1 (uniform distribution), and a random initial phase (uniform distribution) were implemented. As before, I assumed the same linear relationship between stimulus and Poisson inputs *λ*_*A*_ and *λ*_*B*_ and used the default model settings.

The model revealed an interesting relationship between the amplitude of alpha oscillations and the discriminatory power of the decision system. Figure 8e shows the percentage of correct decisions over 2500 ms after stimulus onset for all model runs as a function of amplitude for the threshold value of 50 Hz and five contrast levels. In the range of 0–0.06, the discriminatory power of the system increased with the amplitude. The relationship was significant (probit regression, est. = 12.7, t = 3.3273, *p *= 0.0008769; est. = 9.9, t = 3.4922, *p *= 0.0004791; est. = 7, t = 3.1492, *p *= 0.001637; est. = 6.5, t = 3.3897, *p *= 0.0006996 for contrast levels 13.53, 10.54, 8.21, and 4.98%, respectively) except for contrast level 6.39% (probit regression, est. = 3, t = 1.4631, *p *= 0.1434). The percentage of correct decisions reached its peak at amplitudes in the range 0.05–0.06 (83.2% ± 2.2 SEM; 85.7% ± 2.1 SEM; 94.4 ± 1.3 SEM; 97.3% ± 0.9 SEM; 99.7% ± 0.3 SEM for contrast levels: 4.98, 6.39, 8.21, 10.54, and 13.53%, respectively). For all contrast levels, this peak was higher versus the baseline scenario with no alpha oscillations (75.1% ± 0.8 SEM; 80% ± 0.7 SEM; 86.2% ± 0.6 SEM; 91.3% ± 0.5 SEM; 95.7% ± 0.4 SEM). This was interesting because it showed that the existence of alpha oscillations can improve the discriminatory power of a decision system. After the peak, the discriminatory power dropped because of an increase in the percentage of no-decisions. When the amplitude was in the range of 0.08 to 0.1, alpha waves (frequently) stabilized the system in the spontaneous state and suppressed its ability to converge to a decision (within the predefined period of 2500 ms following stimulus onset). For example, the system maintained the spontaneous state in 39% (± 2 SEM) of cases for the medium contrast level 8.21%. However, this level of no-decisions is not observed behaviorally, which suggests that amplitudes in the range of 0.08 to 0.1 are beyond the levels accompanying decision making in vivo. This level of no-decisions was also very different to that in model runs without or with low amplitudes of alpha waves when the system generated a non-response rate at the level below 2–3%.

In the context of the negative impact of alpha power on the baseline firing rate and the speed of the integration process, it was not surprising to see the negative relationship between the amplitude of alpha oscillations and RT (Fig. 8f). For example, the mean RT increased in the range of 50 ms when the amplitude changed from 0.01 and 0.06 for the threshold value of 40 Hz (linear regression, slope = 849.3, t = 3.898, *p *= 0.000101; bootstrap estimate of slope: 848.56 ± 224.29, 95% confidence interval: (407.26, 1285.83), N = 10,000 draws with replacement).

Model predictions were consistent with most of the experimental findings. Spiking activity in the model was negatively correlated with alpha power, and (by design) spiking activity was phase dependent (as indicated by Haegens and colleagues^50^). Lower spiking activity is connected with a decrease in metabolic rate. Thus, the model predicts the negative correlation between alpha power and the metabolic rate which was observed experimentally in some brain areas^51–54^. However, the experimental data reveals that this effect is not consistent across all brain areas^51–55^. The impact of alpha power on model performance was non-linear: higher alpha power increased the discriminatory power up to the optimal level and then suppressed the ability to make decisions after crossing the optimal level. This is consistent with the studies^39–41^, which showed that a large alpha power during the anticipation of stimulus onset is positively correlated with performance, provided the amplitude of alpha oscillations is at or below the optimal level. This is, however, inconsistent with the experiment of Haegens and colleagues^50^, which showed the opposite effect. Evidence for the relationship between alpha power and decision time was provided by Paluch, Jurewicz, and Wróbel^42^. The model predictions were directionally consistent with this relationship.

### Reconciling the threshold hypothesis with neurophysiological data

The analysis presented in this paper supports the threshold hypothesis of SAT and provides counterarguments against the gain modulation hypothesis. However, the threshold hypothesis alone does not explain the phenomena observed in neurophysiological studies: First, the threshold firing rate of neurons measured experimentally is the same during the speed and accuracy conditions. Second, the baseline firing rate of neurons is higher in the speed condition compared to the accuracy condition. Third, the speed of neuronal integration (measured by modeling the drift rate) is faster during the speed condition compared to the accuracy condition.

**Figure 9:**
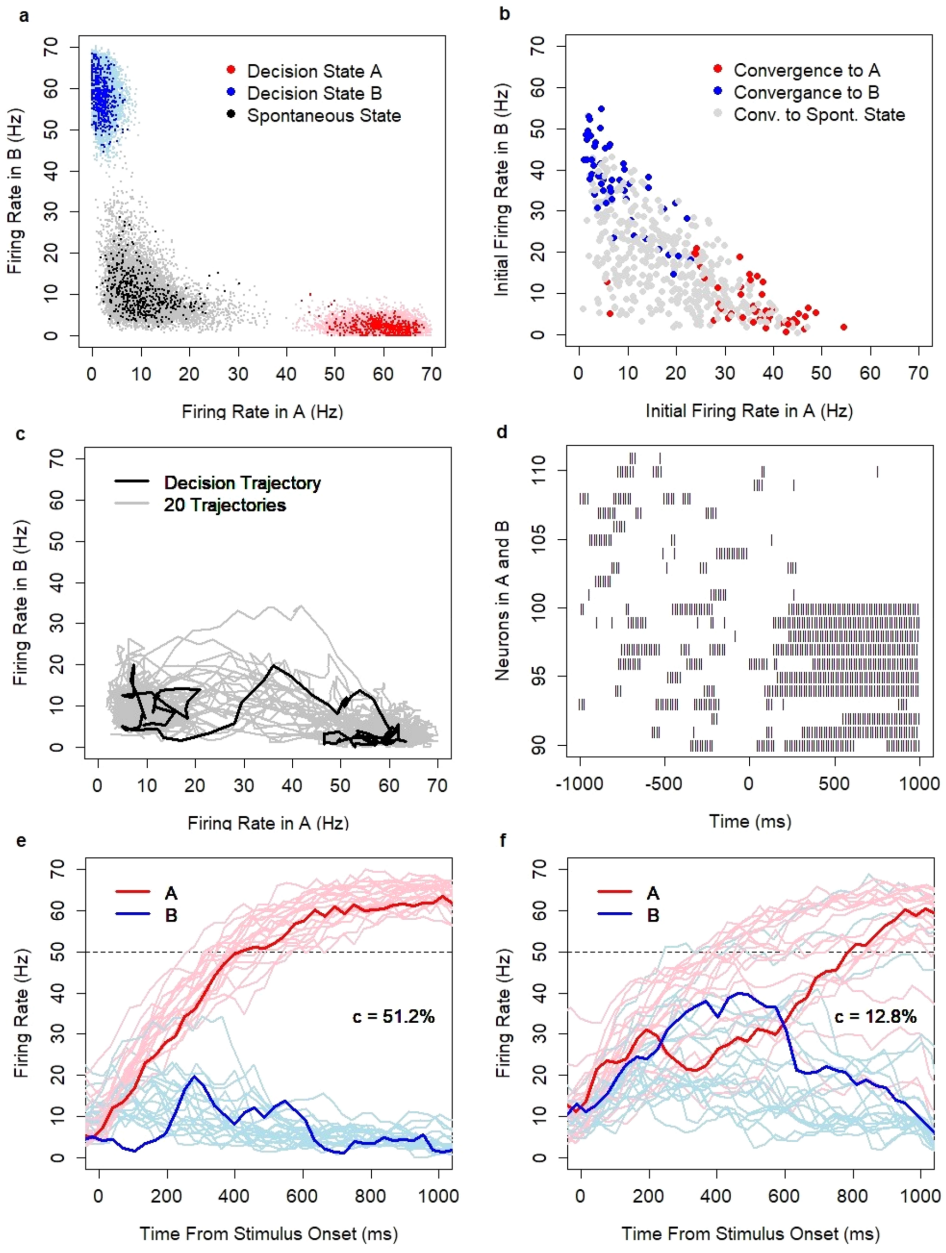
(**a**) The attractor states are the spontaneous state (black) and decision states A (red) and B (blue); N = 1500 data points (bold color), N = 30,000 data points (light hue). (**b**) Basins of attraction: convergence to a steady state within 1000 s from a randomly defined initial state measured within 50 ms after network initialization. (**c**) Decision trajectories for model runs with coherence level c = 51.2%. (**d**) Spiking activity of neurons in pools A (#90–100) and B (#101–111) before stimulus onset (− 1000–0 ms) and after providing input to pool A only (0–1000 ms). (**e**) Firing rate in pools A and B for model runs with coherence level c = 51.2% and threshold value indicated by the dotted line. (**f**) Firing rate in pools A and B for model runs with coherence level c = 12.8% and threshold value indicated by the dotted line. Thicker lines (in **c**, **e**, and **f**) show a single model run selected from a wider range of model runs indicated by thinner lines.

Here I show how these phenomena can be explained assuming the threshold hypothesis. First, notice that the implementation of the threshold hypothesis in terms of population-average firing rate in the decision pool was not inconsistent with a fixed threshold value for neurons in the pool. Neurons in the decision system exhibited a phase transition between the low-frequency state (with a spike-count rate in the range of 10 Hz) and the high-frequency state (with the spike-count rate of 70 Hz assumed in the model). All neurons that were in the high-frequency state showed the same firing rate at the termination of the decision process regardless of the threshold level. However, the transition to the high-frequency state happened at various moments for different neurons (see Fig. 9d in Methods). Hence, the model distinguishes between the neuronal integration process which happens at the level of a population of neurons and the phase transition process which happens at the level of individual neurons. If the same happens in vivo and individual neurons exhibit a phase transition between low and high-frequency states, a lower threshold level would be manifested by a lower coherence between the firing rates of neurons at the moment of a decision, but not by a lower firing rate of individual neurons. The interference of alpha oscillations with the decision process suggests that there is an optimal alpha amplitude level that enhances the decision system’s discriminatory power over the baseline level without alpha oscillations. The fitness maximization principle implies that this amplitude of alpha oscillations should be maintained in the accuracy condition. Conversely, alpha oscillations should be suppressed during the speed condition because higher amplitude levels imply longer RTs. This suggests that the magnitude of alpha waves suppression during ERD of alpha oscillations is lower for the accuracy condition and higher for the speed condition. Higher magnitudes of alpha power suppression result in an increased baseline firing rate and an increased speed of neuronal integration. Hence, the increased baseline firing rate and the increased speed of neuronal integration accompany the speed condition, as observed experimentally^17,24^. The increased spiking activity corresponds with the increased hemodynamic response^18–22^. Thus, the interaction of alpha oscillations with the decision process explains the neurophysiological data related to differences between the speed and accuracy conditions.

Note that in all simulations presented in this paper, I have assumed that the amplitude of alpha waves was constant and applicable throughout the entire decision process including the pre-decision time and neuronal integration time. This was sufficient to show the relationships between the alpha oscillations and the decision process but did not reflect the dynamic mechanism of ERD observed in vivo.

## Discussion

Investigations of alternative computational mechanisms of SAT based on the behavioral experiment of Rafiei and Rahnev^43^ and using the recurrent attractor network model with binary neurons^45^ led to the conclusion that SAT is controlled by threshold adjustments in the choice circuit performing the neuronal integration process. Our results excluded the possibility that SAT is controlled through the mechanism postulated by the gain modulation hypothesis^28^.

The gain modulation hypothesis explains the neurophysiological evidence, which shows an increased baseline firing rate and an increased speed of neuronal integration during the speed condition by postulating that these effects are related to the increased excitability of neurons in the choice circuit. This evidence comes from electrophysiological studies^17,24^ and is consistent with the results of neuroimaging studies^18,22^, which show an increased hemodynamic response during the speed condition. However, computational analysis supporting the gain modulation hypothesis does not explain the shorter RTs on erroneous than correct decisions in the speed condition^23^. The evidence for shorter RTs on errors in the speed condition comes from the Rafiei and Rahnev experiment^43^ and previous research^36,37^. Our analysis showed that the ability of the attractor network computing categorical choice to produce shorter RTs on erroneous decisions was threshold-related, and neither manipulations of inputs nor excitability of neurons in the network facilitated consistently shorter RTs on errors. Thus, the ability of the decision system to produce shorter RTs on errors is a property which can be used to distinguish between alternative computational mechanisms of SAT and leads to the conclusion that SAT is implemented through threshold adjustments.

The experiment of Rafiei and Rahnev^43^ studied SAT effects under multiple SAT conditions and showed non-linear robust U-shape patterns with regard to the RT difference between erroneous and correct decisions, the SD/mean RT ratio, and RT skewness as a function of SAT condition. Our computational analysis revealed that all these patterns were qualitatively consistent with the threshold hypothesis and were inconsistent with the gain modulation hypothesis. Thus, the computational evidence in favor of threshold adjustment as the control mechanism of SAT goes beyond the argument related to shorter RTs on errors.

The decision variable was defined in terms of the population-average firing rate in the winning attractor, whereas the dynamics of individual neurons were best described in terms of the phase transition between the low-frequency state (with spike-count rate in the range of 10 Hz) and the high-frequency state (70 Hz assumed in the model). This implied that all neurons participating in the trial-to-trial neuronal integration process showed the same firing rate at the termination of the decision process regardless of the threshold level. This is consistent with neurophysiological evidence, which shows the same firing rate of neurons for the speed and accuracy conditions. If the same happens in vivo and individual neurons exhibit a phase transition between low and high-frequency states, a lower threshold level would be manifested by a lower coherence between the firing rates of neurons at the moment of decision but not by a lower firing rate of individual neurons. The ergodicity of the system implies that the decision variable can be represented in the firing rate of individual neurons averaged over multiple trials. Hence, the threshold hypothesis is not inconsistent with the experimental evidence.

However, the threshold hypothesis alone does not explain the increased baseline firing rate and the increased speed of neuronal integration in the speed condition. Our results suggest that these effects could be linked to the event related desynchronization (ERD) of alpha oscillations (8–12 Hz)^38^, which interfere with the decision process. Simulations of the impact of alpha oscillations on the decision process revealed the increased decision time as a function of the amplitude of alpha oscillations and predicted the existence of an optimal amplitude of alpha oscillations that enhanced the discriminatory power of the decision circuit. This suggests that the magnitude of alpha waves suppression during ERD depends on the SAT condition in vivo. In the accuracy condition, alpha oscillations are maintained to enhance the discriminatory power of the decision system. In the speed condition, alpha oscillations are suppressed to decrease the reaction time. At the neuronal level, the model predicts that suppressing alpha oscillations leads to an increase in the baseline firing rate and an increase of the speed of neuronal integration, which are observed experimentally during the speed condition^17,24^.

The model predictions were consistent with most of the experimental evidence. Several studies^39–41^ have shown that large alpha power during the anticipation of stimulus onset is positively correlated with performance. The model revealed the non-linear impact of alpha oscillations on decision accuracy: higher alpha power increased discriminatory power up to the optimal level and then suppressed the ability to make decisions after crossing the optimal level. Thus, the model predictions were consistent with these experiments when alpha power was at or below the optimal level. Paluch, Jurewicz, and Wróbel^42^ analyzed the responses among groups of participants responding fast or slowly to a decision task and showed that those responding fast had a lower alpha power in the period preceding stimulus onset. Model predictions for decision time were directionally consistent with this experiment. The experiment of Haegens and colleagues^50^ revealed a negative relationship between alpha power and the spiking activity of neurons. This was consistent with model predictions. Several neuroimaging studies^51–54^ have reported a negative correlation between metabolic rate and alpha power. The observed effects were, however, inconsistent between brain regions. The experiment of Haegens and colleagues^50^ showed that a decrease in alpha power (in premotor and motor regions) was correlated with better discrimination performance. This was inconsistent with model predictions.

Model simulations revealed the interference of alpha waves (8–12 Hz) with the decision process at the behavioral and neuronal levels. These model predictions can be tested in future experimental research. Further modeling effort is also required. In all current simulations, the amplitude of alpha waves was constant and applicable throughout the entire decision process, including the pre-decision time and neuronal integration time. However, experimental data^38^ shows that the desynchronization of alpha oscillations (i.e., ERD) is a dynamic process. Investigating these dynamics was beyond the scope of the current paper.

Our analysis narrowed the possibilities for SAT mechanisms investigated previously. Several studies have reported modulation of evidence encoding and an increased drift rate^17,25,56^ in the speed condition. In our model drift rate is controlled through the strength of inputs. However, no manipulation of input strength could lead to shorter RTs on erroneous decisions than correct ones. Thus, our results support the current view that the modulation of evidence encoding is not a causal mechanism of SAT. The same argument applies to potential additional sources of input in addition to the inputs related to incoming evidence. Such (or equivalent) mechanisms have been considered by Ditterich^57^ and Cisek and colleagues^58^.

Mechanisms of controlling SAT via an urgency signal were discussed by Murphy and colleagues^29^, who postulated a global gain modulation mechanism with time-dependent urgency. This hypothesis is broader and goes beyond the study of computational schemes through which the choice system is affected. However, our analysis could be informative about how the postulated urgency signal would control the performance of the choice system. Our results supported the possibility that the urgency signal controls SAT by adjusting the decision threshold but excluded the possibility that it controls SAT by modulating either inputs or the excitability of neurons in the choice circuit.

Note that our conclusion that SAT is controlled by threshold adjustments might be limited to the reward scheme of manipulating time pressure used by Rafiei and Rahnev^43^ if the underlying brain mechanism of SAT depends on a scheme by which time pressure is manipulated. Recent experiments^59^ showed that cue-based and deadline-based schemes of manipulating time pressure in experimental research can result in different behavioral responses, which are best explained by threshold level and collapsing bounds, respectively.

A large body of research^59–64^ has investigated the possibility that decision thresholds are dynamic (i.e., collapsing bounds). Collapsing bounds are implemented through time-variable thresholds in the modeling practice, which involves system-level models of decision making, and are sometimes interpreted as urgency signals. Some of these considerations^58^ go beyond the sequential sampling paradigm considered here. These studies provide mixed evidence in favor of collapsing bounds or urgency signals. However, if the brain can flexibly set the fixed threshold, as suggested by the current study, it is plausible that it can also adjust the threshold during decision making. This possibility was not investigated in the current paper. All model simulations involved fixed threshold levels, which reflected the experimental paradigm used by Rafiei and Rahnev^43^.

Ratcliff and Kang^44^ argued that the data of Rafiei and Rahnev^43^ were contaminated by fast responses that did not come from the decision processes and were instead fast guesses. If they are correct, our argument based on fitting U-shape data patterns would be invalid. However, the main argument that shows that shorter decision times on errors are related to threshold adjustment and that this property can be used to distinguish between alternative computational mechanisms of SAT remains valid. Empirical evidence for shorter decision times on errors in the speed condition comes from several experiments^36,37,43^. Our modeling approach also suggests that fast guesses can be performed by the same neuronal mechanism that computes decisions.

Our findings suggest that the accuracy condition is the default mode of decision making. In the accuracy condition, the threshold is defined objectively as the border of the basin of attraction of the network’s decision state. This does not apply to the speed condition for which the threshold level is discretionary within a reasonable range. Several authors^20,22^ have previously suggested that the accuracy condition is the default decision-making mode. Van Maanen and colleagues^22^ studied neuronal correlates of trial-to-trial variability of behavioral responses and found that the hemodynamic responses in the presupplementary motor area and striatum were correlated with response variability, which could be attributed to the threshold level of the underlying decision process (or response caution in the terminology used by the authors). Interestingly, this correlation applied only to the speed condition. This aligns with our view because trial-to-trial variability in setting the threshold level in the speed condition would translate into measurable fluctuations of behavioral response. Wheras trial-to-trial variability in the accuracy condition is stochastically driven.

The question of how the threshold hypothesis is mechanistically implemented in neural circuits requires further investigation. The idea of distributed decision systems^28,31,65,66^, however, provides a suggestion for a plausible direction of the future research. Under the threshold hypothesis, SAT is an inference problem of whether the ongoing neuronal integration process will eventually converge to a decision state. The idea of an observer of the choice system was previously discussed by Simen^67^. Influential papers^11,15^ provided compelling arguments in favor of the thesis that the brain uses an integration-to-the-bound process to solve such inference problems. If so, the inference problem of the SAT might be mechanistically implemented as a downstream neuronal integration process. The upstream choice process is located in the lateral intraparietal (LIP) area^8,9,17^ for decisions involving eye movements. The downstream process triggers a behavioral response and is located in the superior colliculus^68–70^. Inputs to the downstream process are gated through tonic inhibition in basal ganglia. One can speculate that the gating inhibition level in basal ganglia modulates the transfer of the signal between these two parallel processes and this modulation sets a SAT condition. When accuracy is a priority, a higher gating inhibition level results in a (default) weaker signal and slower neuronal integration of the downstream process. When speed is a priority, a lower gating inhibition level results in a stronger signal facilitating faster neuronal integration of the downstream process. The termination of the downstream process enforces the termination of the parallel upstream choice process. In the speed condition this termination happens ahead of the upstream choice process reaching the decision (attractor) state of the network performing a choice computation. The inhibitory role of basal ganglia for the implementation of SAT in the brain was previously suggested by many authors^16,20^. Several studies^19–22,71^ have provided empirical evidence of the involvement of a cortico-basal ganglia circuit in controlling the SAT.

All results presented in this paper were obtained by simulating the decision-making model based on an attractor network with binary neurons^45^. The current model showed neuronal dynamics that were consistent with neurophysiological evidence. It replicated the dot motion discrimination experiments^8,9^ with realistic predictions related to accuracy and response time. In this study, I showed that the model was also qualitatively consistent with the experiment of Rafiei and Rahnev^43^ and was able to replicate the demanding U-shape patterns under the threshold hypothesis. The current study provided several new predictions that can be tested in future experimental research and can confirm or falsify the proposed modeling approach. In particular, the current study suggests that the interference of alpha oscillations with the decision process is a non-negligible factor. Future experimental studies and modeling approaches should account for this interference.

## Methods

The results presented in this paper were obtained in silico through an attractor network model with binary neurons^45^. The last two decades of neurophysiological and theoretical research^6–9,11,12,34,35,46^ have led to establishing the paradigm of how decision making for a two-alternative forced-choice task is implemented in the brain. The neural circuit computing a categorical decision is a recurrent network with global inhibition, which facilitates winner-take-all competition between the attractors. Wang developed the mechanistic implementation of a decision-making circuit with leaky integrate-and-fire neurons^34^. An alternative implementation of the circuit with binary neurons^45^, considered in this paper, is based on the following four phenomenological assumptions:

### Neurons

Integrate inputs from their presynaptic connections, and when the threshold is reached, they fire an action potential. After emitting a spike, they stay inactive over the refractory period. In the model, a binary state is assigned to each neuron (j = 1, … N, where N = 1000) at time *t*: *s*_*t*_(j) = 1 if the neuron is in the refractory period and 0 otherwise. A spike is emitted each time the neuron is updated to state *s*_*t*_(j) = 1. For simplicity of implementation, I assumed that the length of the effective refractory period is the same as the synapse constant d (i.e., the time of integrating inputs). With this convention, the excitatory input from presynaptic neurons to neuron j is given by:

$${\int }_{t-d}^{t}{\sum }_{i=1}^{N}\delta (\tau -{t}_{k}\left(\mathrm{i}\right)){w}_{\mathit{ij} }\mathit{d\tau } = {\sum }_{i=1}^{N}{s}_{t}\left(\mathrm{i}\right){w}_{\mathit{ij} }$$

where *t*_*k*_(i), k = 1, 2, … is the sequence of times at which spikes occur in the presynaptic neuron i, and *w*_*ij*_ (i = 1, .. N) are the presynaptic connections of the neuron j. The integration is taken over the period (*t*−*d*, *t*) where *τ* is the integration time variable, and *δ* is the Dirac delta function used to describe the spiking activity of neurons. Thus, the presynaptic input on the left-hand side is determined by the states *s*_*t*_(j) of neurons at time *t* and connection weights *w*_*ij*_ as given by the right-hand side of the equation. Connections are random and binary, that is *w*_*ij*_ = 0 or 1. No restrictions on the connections are imposed. In particular, the network is not assumed to be symmetric.

### Global inhibition

Ensures the stability of the network with a balance between excitation and inhibition and facilitates winner-take-all competition between decision states. Global inhibition is implemented through a common inhibitory threshold in the model. With balanced excitation and inhibition, the threshold is a function of the activity of excitatory neurons, and the model can be reduced to excitatory neurons only. I postulate that the threshold is set in relative terms and varies with the square of the proportion of active neurons during the update time. The update rule is given by:

$${s}_{t}\left(\mathrm{j}\right)= 1\mathrm{ iff}\;\frac{{\sum }_{i=1}^{N}{ s}_{t}\left(\mathrm{i}\right){w}_{\mathit{ij} } }{{\sum }_{i=1}^{N}{w}_{\mathit{ij} } }> \frac{1}{\Theta }{\cdot \left(\frac{{\sum }_{i=1}^{N}{ s}_{t}\left(\mathrm{i}\right) }{N}\right)}^{2}$$

where *Θ* is the inhibitory constant (*Θ* = 0.13), which defines the normative excitation level of neurons in the system. The proposed update rule ensures a persistent activity in the absence of exogenous stochastic stimulation, provided the initial state of the network is non-zero. The model is initialized with random non-zero states with probability *Θ*.

### Asynchronous evolution

Of the network follows the Poisson point process^72,73^, namely the time intervals between consecutive updates are distributed exponentially with rate *τ*_*2*_ (*τ*_*2*_ = 0.006) except for the constant refractory periods 1/*τ*_*1*_ ~ 14.3 ms (*τ*_*1*_ = 0.07); the latter sets the maximum firing rate (70 Hz) of neurons in the model. Note that the assumed refractory period is longer than the observed refractory period of neurons in vivo. The assumed constant reflects the effective time interval between two consecutive spikes emitted by neurons when they fire with the maximum firing rate.

### Network architecture

Consists of three pools of neurons: two decision pools, A and B (each n = 100), with a higher density of random connections within each set *d*_*1*_ (*d*_*1*_ = 0.55), and the rest of the network. Connections in the rest of the network and between different pools of neurons have density *d*_*2*_ (*d*_*2*_ = 0.36). A higher density of connections in the decision pools is assumed to be the result of Hebbian learning^74^; no learning mechanism is introduced in the model. Connections are regenerated before each model simulation and are kept constant during a simulation.

The decision sets A and B are selective to inputs. Inputs are latent variables which reflect the stimulation of the decision system by a pool of sensory encoding neurons reacting to stimuli. This stimulation increases the excitatory postsynaptic input delivered to neurons in the decision system, in addition to the input provided by recurrent connections as defined by the left-hand side of the update rule. The strength of inputs is assumed to be directly related to the stimulus (linear relationship) and is delivered in the form of time-variable sequence of spikes. This sequence (of natural numbers) is derived from the Poisson distribution (Poisson inputs) with the parameters *λ*_*A*_ and *λ*_*B*_ reflecting the strength of the evidence in favor of decisions A and B, respectively. Alternatively, inputs can be non-random and provided by time-constant real values that modify the excitatory input to neurons in A and B. Non-random inputs are not biophysically realistic, but they produce results equivalent to those with the Poisson inputs. Inputs are provided to 50% of neurons in A and B, respectively.

The model has several control parameters: the size of network (N = 1000) and pools A, B (n = 100 each), the inhibitory constant (*Θ* = 0.13), parameters controlling the time evolution of the network (*τ*_*1*_ = 0.07 and *τ*_*2*_ = 0.006), and the density constants (*d*_*1*_ = 0.55 and *d*_*2*_ = 0.36). The control parameters are responsible for the emergent attractor dynamics of the system. Some model parameters are arbitrary (e.g., its overall size and the sizes of the decision pools A and B). For the inhibition mechanism to facilitate winner-take-all competition , the value of the inhibition constant *Θ* falls into the range from 0.1 to 0.2. Parameter *τ*_*1*_ = 0.07 defines the maximum frequency of spikes (70 Hz). This value is consistent with the maximum firing rate of lateral intraparietal (LIP) neurons in the human brain^46^. In the version of the model considered in this paper, we assigned a constant time 1/*τ*_*1*_ ~ 14.3 ms (*τ*_*1*_ = 0.07) to neurons in the refractory period, while in the previous version^45^, the refractory period was assumed to be random (exponential distribution). It was confirmed that both choices led to the same emergent dynamics and comparable model performance. Parameter *τ*_*2*_ (*τ*_*2*_ = 0.006) controls the speed of neuronal integration and was set experimentally in order for the model to produce biophysically realistic RTs (in the range of 100 s of ms).

With the predefined values of the parameters (N, n, *Θ*, *τ*_*1*_, and *τ*_*2*_), the model works only under a particular and narrow range of density ratio *d*_*1*_/*d*_*2*_^45^. If the ratio is greater than this value, the model becomes unstable and converges to a decision within a short amount of time with no inputs provided. If the ratio is smaller than this value, the model becomes insensitive to inputs and maintains the spontaneous state despite inputs. The control parameters were fixed at their default values if not stated otherwise.

The model has three attractor states (Fig. 9a): the spontaneous state with a low firing rate 4–20 Hz (mean firing rate in the decision pool: 10.3 Hz, SD = 5.2) and two decision states when the vast majority of neurons in the winning decision set (either A or B) fire with a high firing rate 51–66 Hz (mean: 59.7 Hz, SD = 4.7). When the model is in one of the decision states, the firing rate in the losing pool is suppressed to 0–5 Hz (mean: 2 Hz, SD = 1.4). With no inputs, all three steady states are semi-stable in the sense that the model maintains each state for a period of time, but the chance of escaping from the state is non-zero, so the system eventually moves to another state. The estimated median time to escape from the spontaneous state is in the range of 35 s (35,000 ms), and the escape dynamics follow an exponential decay (estimated relaxation time: 50 s). The estimated median time to escape from the decision state is in the range of 10 s. Interestingly, the process does not follow an exponential decay. The leak rate is not constant and diminishes over time. The semi-stability of the decision states ensures the ability of the model to serve as a working memory system. The boundaries of basins of attraction are fuzzy (Fig. 9b); a similar randomly chosen initial state can converge to a different steady state. This effect signifies that the model behaves chaotically.

When inputs are provided, the system moves from the spontaneous state to one of the decision states (Fig. 9c). The spiking activity of neurons is consistent with the neurophysiologic observations. In the spontaneous state, neurons fire irregularly and generate bursting activities (Fig. 9d, time: − 1000–0 ms). When an input is provided to one of the decision sets, the neurons in this set switch from a low firing rate to a high firing rate while neurons in the opposite set are silenced (Fig. 9d, time: 0–1000 ms). The system facilitates probabilistic decision making. The probability of reaching the decision state increases with the stimulus strength and stimulus duration while the time to reach the decision state decreases^45^. The ramping activity of neurons and the speed of neuronal integration depend on the relative strength of inputs to both decision sets. In dot motion discrimination experiments, task difficulty is controlled by the value of coherence level (denoted by c). In an easy task with a higher difference between inputs (Fig. 9e, coherence c = 51.2%), the system converges to the correct decision in most cases, and the RT is short. In a difficult task with a lower difference between inputs (Fig. 9f, coherence c = 12.8%), the system often converges to erroneous decisions, and the RT on correct decisions is longer. For simplicity of notation, I denote by A and B both the decision sets (i.e., pools of neurons in the system) and the decision states (i.e., states of the entire system when the pools A or B fire with high frequency).

Model predictions were validated based on the dot motion discrimination experiments^8,9^. In these experiments, monkeys reacted to a display of dots moving coherently to one of two possible directions among a broader set of randomly moving dots. The task was to identify the direction, and the difficulty of the task depended on the coherence level c (i.e., the percentage of dots (0–100%) moving coherently to one of the directions). The experiments were conducted in two distinct decision paradigms: the working-memory (fixed duration) paradigm when the subject was requested to provide a response after a fixed (randomized) time after the stimulus expired or the reaction-time paradigm when the subject provided a response after stimulus onset whenever ready. In these experiments, neurons in the LIP area displayed a ramping to threshold activity consistent with the monkey’s behavioral response. Experimental data have revealed the characteristic relationship between the coherence level and the decision accuracy (percentage of correct decisions), which follows the Weibull psychometric function:

$$\% correct=1-\frac{1}{2}*\mathrm{exp}(-{\left(\frac{c}{\alpha }\right)}^{\beta })$$

where c is the coherence level, and α, β are parameters. The shape of the relationship is consistent between experiments and decision paradigms. The estimated parameters α and β vary: α = 15, β = 1.1 in the Shadlen and Newsome experiment and α = 6, β = 1.7 in the Roitman and Shadlen experiment (as reported by Wang^34^). Under the reaction-time decision paradigm, the response time increases with task difficulty and is shorter for correct decisions and longer for erroneous decisions across all coherence levels (c > 0).

**Figure 10:**
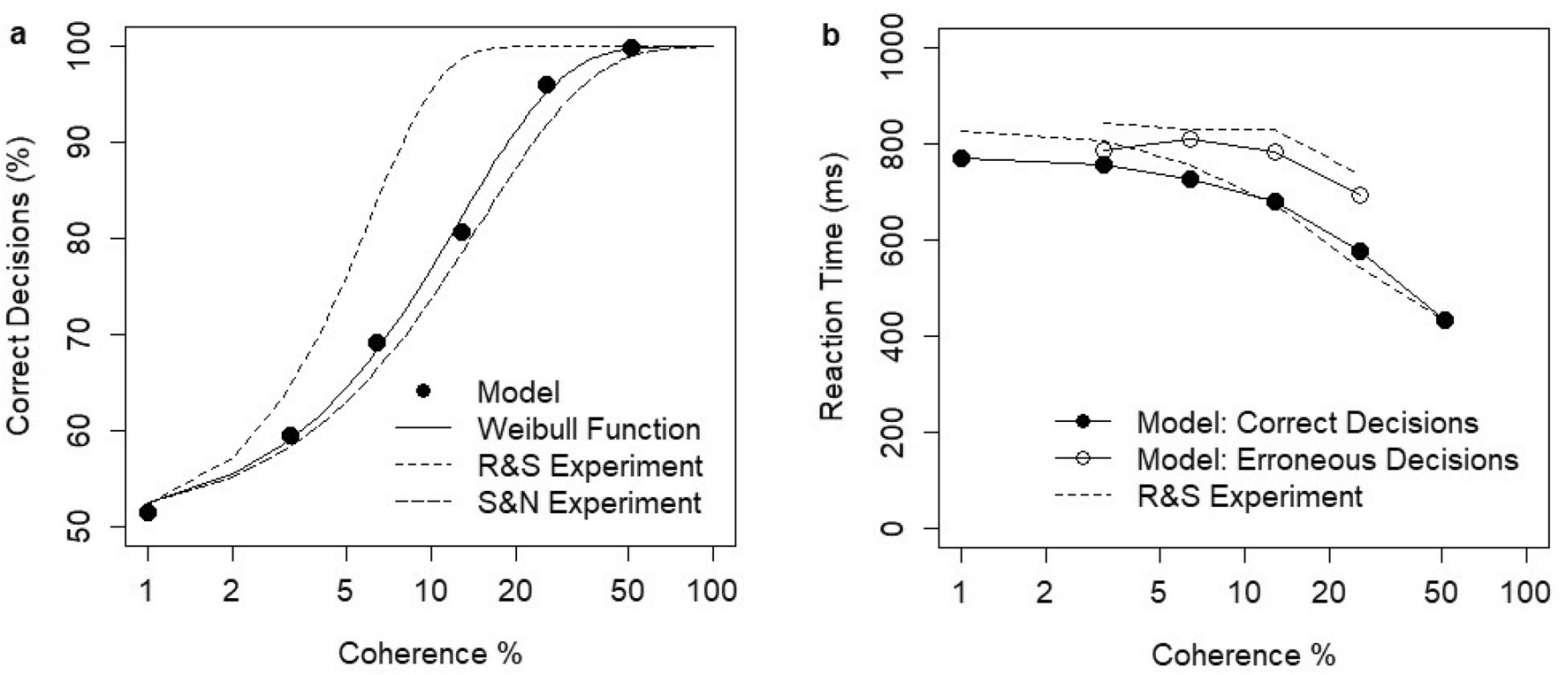
Model predictions for the dot motion discrimination task. (**a**) Psychometric function: N = 12,000 model runs (2000 per coherence level c = 0, 3.2, 6.4, 12.8, 25.6, and 51.2%), SEM: 0.09–1.14; dotted lines: approximation of the experimental data of Shadlen and Newsome (S&N) ^8^ and Roitman and Shadlen (R&S) ^9^ with the Weibull function; solid line: approximation of model estimates: α = 12.5 and β = 1.18. (**b**) Reaction time (RT) as a function of correct and erroneous decisions and coherence level, SEM: 2.5–45.3; dotted lines: experimental data of Roitman and Shadlen (R&S) ^9^, the weighted average from two monkeys. Shadlen and Newsome ^8^ used the working-memory (fixed duration) paradigm only, and hence RT was not analyzed.

Model predictions were consistent with the experimental data. Decision accuracy followed the psychometric function with realistic parameters: α = 12.5, β = 1.18 (Fig. 10a). Mean RTs fitted the observed patterns and produced realistic values (Fig. 10b). The absolute difference between estimated RT and the experimental data^9^ (the weighted average from two monkeys) was in the range from < 1 to 58 ms (the discrepancy of 58 ms related to erroneous decisions with coherence level 3.2%). The average discrepancy between model predictions and the experiment was 35 ms (4.6%) across correct and erroneous decisions and various coherence levels.

The above results were based on N = 12,000 simulations (2000 per coherence level) using standard values of the control parameters. Poisson inputs *λ*_*A*_ and *λ*_*B*_ (sampled every 30 ms over a period of 2 s after stimulus onset) were assumed to be linearly dependent on the coherence level c = 0, 3.2, 6.4, 12.8, 25.6, and 51.2%:

$${\lambda }_{A}= {\lambda }_{0}\frac{1+c}{2}$$

$${\lambda }_{B}= {\lambda }_{0}\frac{1-c}{2}$$

where *λ*_*0*_ = 15. The linear relationship between stimulus and inputs to the decision system was in-line with previous practice^34,35,75^. This practice was based on the observation that the firing rate of MT neurons (providing inputs to the sensory decision system in the LIP area) was a linear function of motion strength. Stimulus onset followed 1000 ms of spontaneous model run. The decision threshold was set at 52.5 Hz. The firing rate was calculated based on 30-ms lags sampled every 10 ms. No-decisions, decisions that took longer than 2000 ms, and decisions made earlier than 100 ms after stimulus onset were removed (2.5%). No non-decision time was assumed.

The model exhibited a non-linear integration dynamics consistent with the experiments^48^. The sensitivity of the decision variable to changes in inputs decreased with the amount of integration and time from stimulus onset (unpublished).

The model discussed here is conceptually similar to Wang’s model^34^. Both models use recurrent attractor networks with a global inhibition mechanism and attractor sets consisting of neurons with a higher density of connections within the sets. However, the models differ in many significant ways. Wang’s model is built with leaky integrate-and-fire neurons, which describe the physical properties of the depolarization process through such parameters as the membrane potential or the current provided by different types of synapses. The model discussed here uses binary neurons that record spikes emitted by the neurons but it does not aim at modeling the depolarization process or the physical properties of the neurons. Wang’s model consists of excitatory and inhibitory neurons, while the current model uses a global (time-dependent) threshold to implement the global inhibition mechanism through an update rule. This allows the network of neurons to be reduced to excitatory neurons only. The update rule used in the current model ensures the persistent activity of the network unlike in the case of Wang’s model, which requires external stimulation of its neurons. Despite the differences in the level of detail in which the neurons are described and modeled, both models show similar dynamics.

Both the current and Wang’s models are mechanistic models of decision making. They aim to describe the way a network of neurons computes a decision. Mechanistic models are conceptually different from system-level models such as the drift diffusion model (DDM)^13^ or the leaky competing accumulator model^46^. System-level models postulate a stochastic process that underlies decision making. Although system-level models have been successfully used to study neurophysiological phenomena, they require an interpretation of the stochastic process in terms of variables of the network of neurons performing the decision (usually the process is interpreted in terms of the firing rate of neurons). The parameters of the process are derived from the experimental data, and the data are used to scale the model by fixing some parameter value at a specific level. Other model parameters are then set relative to the chosen value of this parameter. In the mechanistic models (such as the one discussed in this paper), parameter values are neurophysiologically inspired and are set in advance. In a neurophysiological context, using mechanistic models has two main advantages: 1) they interpret the variables and parameters in a direct way, as the model construction itself provides such an interpretation, 2) the mechanistic approach sets constraints on how the stochastic process is physically performed, which are not present in the system-level approach. These constraints are instrumental for uncovering the neurophysiological mechanisms because they help to exclude some possibilities of how a phenomenon under consideration can be implemented in the brain.

## Supplementary Information

Supplementary Information.

## Data Availability

The model was implemented in R^76^ version 4.1.1 (2021-08-10). The datasets generated and analyzed during the current study are available in the Github repository, https://github.com/MarcinPenconek/Computational-Analysis-of-Speed-Accuracy-Tradeoff.
