# Supplementary material for: Computational analysis of speed-accuracy tradeoff
Source: Sci Rep. 2022 Dec 20;12:21995. doi: 10.1038/s41598-022-26120-2 (PMC9768160; doi:10.1038/s41598-022-26120-2)
Supplement: Supplementary file 1 — Supplementary Information. [file 41598_2022_26120_MOESM1_ESM.docx]

APPENDIX: Bootstrap Estimates

The appendix contains bootstrap estimates of the mean RT difference between erroneous and correct decisions for the threshold hypothesis (Table 1) and the gain modulation hypothesis (Table 2). A standard nonparametric bootstrap procedure was conducted and involved drawing N = 10,000 sub-samples with replacement for each data point.

Table 1: Estimates from the total sample (estimate) and bootstrap estimates of mean RT difference (BS mean), standard deviation, and 95% confidence interval (CI) for the threshold hypothesis (frequency threshold: 20–50 Hz) and contrast levels: 4.98, 6.39, 8.21, 10.54, and 13.53%.

| **Estimate**  BS mean (± SD)  CI: 2.5%, 97.5% | **20 Hz** | **25 Hz** | **30 Hz** | **35 Hz** | **40 Hz** | **45 Hz** | **50 Hz** |
| --- | --- | --- | --- | --- | --- | --- | --- |
| **4.98%** | **-3.3**  -3.30 (± 1.34)  (-5.95, -0.71) | **-1.0**  -0.99 (± 2.10)  (-5.11, 3.14) | **-0.5**  -0.47 (± 3.01)  (-6.36, 5.39) | **1.4**  1.38 (± 4.40)  (-7.04, 10.19) | **12.6**  12.50 (± 6.80)  (-0.63, 26.03) | **40.6**  40.51 (± 10.56)  (19.77, 61.42) | **84.4**  84.38 (± 14.76)  (55.52, 113.54) |
| **6.39%** | **-6.6**  -6.59 (± 1.35)  (-9.22, -3.95) | **-9.0**  -9.03 (± 2.14)  (-13.22, -4.79) | **-12.0**  -11.97 (± 3.09)  (-17.96, -5.79) | **-10.8**  -10.81 (± 4.63)  (-19.96, -1.78) | **0.4**  0.40 (± 7.51)  (-14.12, 15.15) | **25.9**  25.97 (± 11.76)  (2.66, 48.90) | **42.2**  42.37 (± 15.15)  (12.85, 72.24) |
| **8.21%** | **-6.5**  -6.52 (± 1.41)  (-9.29, -3.79) | **-9.5**  -9.48 (± 2.18)  (-13.74, -5.15) | **-12.1**  -12.04 (± 3.17)  (-18.19, -5.83) | **-16.3**  -16.28 (± 4.78)  (-25.68, -6.74) | **-14.5**  -14.48 (± 7.86)  (-29.77, 1.04) | **7.3**  7.11 (± 12.60)  (-16.93, 32.26) | **70.5**  70.38 (± 18.77)  (33.85, 107.77) |
| **10.54%** | **-6.8**  -6.80 (± 1.44)  (-9.59, -4.01) | **-9.8**  -9.78 (± 2.31)  (-14.28, -5.16) | **-10.5**  -10.43 (± 3.73)  (-17.79, -3.13) | **-15.7**  -15.76 (± 5.68)  (-26.82, -4.61) | **-5.6**  -5.67 (± 9.61)  (-24.28, 13.67) | **11.8**  11.75 (± 14.18)  (-15.55, 39.89) | **65.4**  65.04 (± 22.81)  (21.43, 110.12) |
| **13.53%** | **-13.6**  -13.64 (± 1.35)  (-16.29, -10.95) | **-17.0**  -17.02 (± 2.26)  (-21.54, -12.61) | **-19.3**  -19.33 (± 3.74)  (-26.58, -11.95) | **-16.7**  -16.72 (± 6.19)  (-29.00, -4.76) | **-18.3**  -18.45 (± 10.84)  (-39.01, 2.94) | **2.1**  2.01 (± 18.90)  (-35.00, 38.45) | **57.1**  56.91 (± 32.83)  (-6.53, 123.78) |

Table 2: Estimates from the total sample (estimate) and bootstrap estimates of mean RT difference (BS mean), standard deviation, and 95% confidence interval (CI) for the gain modulation hypothesis (inhibition level *Θ* in the range 0.1–0.2, reverse order) and contrast levels: 4.98, 6.39, 8.21, 10.54, and 13.53%.

| **Estimate**  BS mean (±SD)  CI: 2.5%, 97.5% | **Θ = 0.186** | **0.179** | **0.164** | **0.150** | **0.136** | **0.122** | **0.114** |
| --- | --- | --- | --- | --- | --- | --- | --- |
| **4.98%** | **-14.6**  -14.64 (± 12.89)  (-39.72, 10.74) | **-2,5**  -2.33 (± 11,12)  (-23.87, 19.43) | **-4.7**  -4.65 (± 11.72)  (-27.61, 18.15) | **17.7**  17.55 (± 12.76)  (-6.81, 42.54) | **49.6**  49.29 (± 13.51)  (22.91, 75.22) | **90.7**  90.51 (± 14.56)  (61.97, 119.06) | **115.4**  115.26 (± 18.07)  (79.87, 150.71) |
| **6.39%** | **-1.0**  -0.98 (± 14.03)  (-28.12, 26.00) | **-0.1**  -0.08 (± 11.91)  (-22.93, 23.17) | **15.6**  15.49 (± 12.88)  (-9.19, 41.34) | **40.7**  40.36 (± 13.87)  (13.47, 67.50) | **66.2**  66.15 (± 15.03)  (36.98, 95.85) | **103.7**  103.81 (± 16.50)  (71.67, 137.01) | **141.6**  141.93 (± 20.86)  (101.05,183.65) |
| **8.21%** | **-11.9**  -11.91 (± 14.34)  (-39.41, 16.95) | **-9.8**  -9.76 (± 13.01)  (-34.94, 15.98) | **-3.9**  -4.08 (± 13.82)  (-30.91, 33.06) | **18.6**  18.55 (± 15.65)  (-12.25, 49.05) | **69.0**  68.91 (± 17.70)  (34.37, 103.26) | **98.3**  98.33 (± 18.38)  (62.69, 133.70) | **134.7**  134.84 (± 25.04)  (86.78, 184.89) |
| **10.54%** | **6.1**  6.32 (± 16.52)  (-25.66, 39.00) | **-4.3**  -4,12 (± 13.74)  (-30.62, 22.90) | **4.6**  4.48 (± 14.57)  (-23.32, 33.06) | **7.6**  7.37 (± 16.74)  (-24.75, 40.60) | **18.0**  17.68 (± 19.90)  (-20.77, 57.27) | **55.0**  54.96 (± 23.23)  (10.83, 101.16) | **78.3**  78.05 (± 29.24)  (22.08, 136.32) |
| **13.53%** | **-75.3**  -75.35 (± 15.99)  (-105.87, -43.86) | **-78.7**  -78.94 (± 14.60)  (-107.11, -49.44) | **-72.4**  -72.53 (± 18.42)  (-107.64, -35.98) | **-11.0**  -11.14 (± 23.95)  (-57.79, 36.53) | **48.4**  48.31 (± 28.45)  (-5.74, 103.71) | **69.2**  69.17 (± 32.24)  (7.64, 133.77) | **82.5**  82.72 (± 39.17)  (6.59, 161.66) |
